# Sources of inequality in “living well” with dementia: an intersectional analysis using a British cohort study

**DOI:** 10.1093/geroni/igag009

**Published:** 2026-01-29

**Authors:** Linda Clare, Laura D Gamble, Anthony Martyr, Martin Knapp, Fiona E Matthews

**Affiliations:** Department of Health & Community Sciences, University of Exeter Medical School, St Luke’s Campus, Exeter, United Kingdom; NIHR Policy Research Unit in Dementia and Neurodegeneration, University of Exeter, St Luke’s Campus, Exeter, United Kingdom; NIHR Policy Research Unit in Dementia and Neurodegeneration, University of Exeter, St Luke’s Campus, Exeter, United Kingdom; Population Health Sciences Institute, Newcastle University, Newcastle-upon-Tyne, United Kingdom; Department of Health & Community Sciences, University of Exeter Medical School, St Luke’s Campus, Exeter, United Kingdom; NIHR Policy Research Unit in Dementia and Neurodegeneration, University of Exeter, St Luke’s Campus, Exeter, United Kingdom; NIHR Policy Research Unit in Dementia and Neurodegeneration, University of Exeter, St Luke’s Campus, Exeter, United Kingdom; Care Policy and Evaluation Centre, London School of Economics and Political Science (LSE), London, United Kingdom; Population Health Sciences Institute, Newcastle University, Newcastle-upon-Tyne, United Kingdom; Institute for Clinical and Applied Health Research, University of Hull, Hull, United Kingdom

**Keywords:** Alzheimer’s, Disparity, Social care, Health care, Neurodegenerative

## Abstract

**Background and Objectives:**

A key aim of national dementia policies is to enable people to “live well” with the condition, but the experience of living with dementia, including access to health and social care services, is markedly affected by numerous sources of socioeconomic disparity. We explored how combinations of these disparities among groups of people with dementia are associated with the capability to “live well.”

**Research Design and Methods:**

We used baseline data from 1,537 people with mild-to-moderate dementia in the British IDEAL cohort (2014–2016). This included personal characteristics, living situation, socioeconomic position, and geographical area, and 3 indices of “living well”—quality of life, satisfaction with life, and well-being. Through regression-tree analyses, we explored how the intersection of factors beyond type of dementia and co-morbidity is associated with subgroups of people with dementia experiencing higher or lower capability for “living well.”

**Results:**

Age, education, living situation, income, and home ownership emerged as the strongest differentiators. Drawing on the concept of precarity, we show how the connections between unequal distribution of resources and personal vulnerabilities lead to an accumulation of pressures and shape outcomes.

**Discussion and Implications:**

These findings from a cohort of people with dementia in a major Western economy represent the tip of the iceberg relative to the full extent of national and especially global inequalities. Addressing the impact of these social inequalities requires a sustained focus on developing and implementing policies that improve equity of access to care and support and increase the potential for “living well.”

Innovation and Translational Significance: It is accepted that numerous sources of socioeconomic disparity affect access to support services for people with dementia, but the impact of unequal distribution of resources on the capability to “live well” with the condition has received less attention. Taking an intersectional approach using regression tree methodology, we demonstrated that age, living situation, and home ownership are significant sources of inequality. In those less advantaged, these disparities intersect and are associated with reduced potential for “living well.” Robust policy solutions are needed to improve equity of access to care and support to address the impact of these social inequalities.

## Background and objectives

Dementia is an umbrella term for a set of long-term progressive neurodegenerative conditions that affect a person’s functioning and for which there are currently no effective disease-modifying treatments. Those affected may live with the condition for many years, requiring increasing support from family, friends, the voluntary sector, and statutory services. In this context, a key aim is to optimize functioning (Bickenbach et al., 2023) and, as outlined, for example, in national dementia strategies (Department of Health, 2009), enable the person to “live well” with the condition. “Living well” means experiencing a good quality of life, together with satisfaction with life and a sense of well-being (Institute of Medicine, 2012). Quality of life, and indeed “living well,” is not simply a matter of managing symptoms, but rests on functioning and experience across the full range of life domains and the contribution of these to psychological well-being (Clare et al., 2019). In pursuit of fairness in policy implementation, the question arises as to whether there are observable disparities among groups of people with dementia in the capability to “live well” and whether such disparities amount to unacceptable inequities by nature of being avoidable, unnecessary, or unjust.

Most of the evidence available in this regard focuses on differences in access to the health and social care systems that form an important part of the context within which people experience dementia. People with dementia are affected by health and social care inequalities in relation to several spheres: personal characteristics such as age, sex, and ethnicity (Pereira et al., 2022; Watson et al., 2023); level of disability, including co-morbidity, functional limitations, and challenging symptoms (Dufour et al., 2023); social capital, including socio-economic status and education (Ferretti et al., 2018; van de Vorst et al., 2016); social situation, including living situation and kin relationship with the family carer if there is one (Clare et al., 2024); and environmental context, including area-level deprivation and geographical location (Ornstein et al., 2022; Watson et al., 2023). A recent rapid review identified 38 sources of health and social care inequality between groups of people with dementia in England, Wales, and Northern Ireland, categorized as relating to age, sex, ethnicity, socioeconomic position, geographical location, technology use, disability, and type of dementia (Hodgson et al., 2023). These sources of health and social care inequality represent intermediate outcomes that can contribute to health inequalities.

Less attention has been given to the way in which inequalities play a part in shaping subjective evaluations of quality of life and related outcomes. One reason for this may be a perceived difficulty in distinguishing possible sources of inequality from the effects of the condition itself (Hodgson et al., 2023), perhaps based on an assumption that poor quality of life is inevitable when living with a progressive neurodegenerative condition. Another reason may be that some potentially relevant objective indicators, such as age, sex, and educational level, are frequently controlled for in statistical analyses, thus masking their salience. However, poor quality of life is not inevitable. Broader gerontological research suggests that, in later life, quality of life and satisfaction with life are shaped by health status, socioeconomic resources, and social connections (Prati, 2022; Sulandari et al., 2024). As many people with dementia are also part of the wider older population, many of the risks they face arise from the same structural inequalities, while other risks may be amplified by cognitive difficulties. Recent work on dementia similarly indicates that well-being reflects interpersonal, psychological, and environmental influences rather than clinical factors alone. For example, independence, meaningful activity, and the availability of appropriate support all contribute to the possibility of living well with dementia, and these opportunities are not distributed equally (Rapaport et al., 2020). In the British IDEAL cohort, differences in quality of life, satisfaction with life, and well-being among people with dementia were evident around the time of diagnosis or at an early stage and showed little change over time (Clare et al., 2022). Clinical features such as reduced functional ability and challenging symptoms were associated with lower quality of life, and the poorer quality of life observed in some diagnostic groups, particularly among people with Parkinsonian dementias, may be linked to the specific symptom profile of those conditions (Martyr et al., 2024). Similarly, having more co-morbid health conditions is directly linked to reduced quality of life (Sabatini et al., 2024). Nevertheless, there is evidence that other sources of inequality also play a part (Martyr et al., 2018). Using IDEAL cohort data to examine disparities and possible sources of inequality arising from individual characteristics or aspects of the person’s context provides evidence that a lower quality of life is linked with living in a more deprived area (Wu et al., 2018), having low social capital (Sabatini et al., 2023), living alone rather than with a spouse (Clare et al., 2024), and being from a non-white minority ethnic group (Victor et al., 2024).

While considering discrete factors is informative, it does not take into account of the way in which varying combinations of individual characteristics and structural or systemic factors can accumulate to create multiple levels of disadvantage where pre-existing inequalities relative to other groups (e.g., due to sex or ethnicity) are exacerbated once dementia develops. For example, older women with dementia are more likely than men or younger people to have few educational qualifications and low income, and to be living alone (Ferretti et al., 2018), but less likely to receive support from services (van Horik et al., 2022). Black people with dementia are less likely to be homeowners and more at risk of declining financial assets than their White counterparts (Kaufman et al., 2020). Qualitative analysis of in-depth interviews with IDEAL cohort participants (Hillman et al., 2023), drew on the concept of precarity (Grenier et al., 2017; Portacolone et al., 2019) to explain how the connections between unequal distribution of resources and personal vulnerabilities lead to an accumulation of pressures and shape outcomes for people living with dementia. An intersectional approach is needed to bring these connections into focus.

The current study constitutes a secondary analysis of publicly available baseline data from the IDEAL cohort. As previous analyses of data from this cohort have demonstrated that quality of life, satisfaction with life, and well-being change little over time, with few longitudinal associations involving other relevant factors (Clare et al., 2022), the present analysis focuses on cross-sectional baseline data. Building on the existing evidence base, we apply an intersectional analytical approach to explore how combinations of socio-demographic, clinical, and contextual factors are associated with inequalities in the ­capability to “live well” among people with dementia.

## Research design and methods

### Design

This study comprises a secondary analysis of baseline data from the IDEAL cohort, a longitudinal study of people living with dementia and carers in Great Britain (Clare et al., 2014). IDEAL is registered with UKCRN (registration number 16593) and was approved by the Wales Research Ethics Committee 5 (reference 13/WA/0405) and the Ethics Committee of the School of Psychology, Bangor University (reference 2014-11684). The present analysis used publicly available baseline data collected between August 2014 and July 2016. The data are held in the UK Data Service archive: https://reshare.ukdataservice.ac.uk/854293/.

### Participants

People with dementia were recruited through UK National Health Service (NHS) research delivery networks across England, Scotland, and Wales. NHS research staff identified and approached potentially eligible individuals by screening clinical records and followed up expressions of interest received via the Join Dementia Research national online portal, a UK-wide service that enables people aged 50 or over to register their interest in taking part in dementia research and being matched to suitable studies. NHS research staff conducted all initial participant contact, eligibility screening, and consent procedures, and undertook all baseline data collection visits. Inclusion criteria at baseline were a diagnosis of dementia made by a clinician at the recruitment site, a score of 15 or above on the Mini-Mental State Examination (Folstein et al., 1975) indicating mild-to-moderate dementia, and the ability to communicate verbally in English. Exclusion criteria were co-morbid terminal illness and inability to provide informed consent. Where possible, a family caregiver was recruited to participate alongside the person with dementia. At baseline, assessments lasted 5 hr and were conducted over three home visits. People with dementia completed questionnaires that were administered face to face by trained researchers, and carers completed questionnaires by themselves, usually in a separate room. A total of 1,537 participants with dementia were recruited at baseline.

### Measures

#### Clinical features

Dementia diagnosis was ascertained from medical records and was classified as Alzheimer’s disease, vascular dementia, mixed Alzheimer’s disease and vascular dementia, frontotemporal dementia, Parkinson’s disease dementia, dementia with Lewy bodies, or other/unspecified dementia. The number of co-morbid conditions (0, 1, or 2+) was derived from responses to the Charlson Comorbidity Index (Charlson et al., 2008). This was administered as a joint interview involving the person with dementia and carer where there was a carer available participating and was completed by the person with dementia where there was no carer available.

#### Potential sources of inequality

Personal characteristics considered were age, sex, marital status, ethnicity, and living situation (alone/with others). Age was grouped into bands (<65 years/65–69 years/70–74 years/75–79 years/80+ years). Marital status was classified as married (married/has a partner/is cohabiting) or other (single/divorced/­separated/widowed). Ethnicity was classified as white (white British/white other) or other (Bangladeshi, Indian, Pakistani, black-African or Caribbean, mixed). Socio-economic position indicators were National Statistics Socio-economic Classification-3 (NS-SEC3) occupational categories (higher managerial, administrative, and professional/intermediate/routine and manual), education (no qualifications/school leaving certificate at age 16/school leaving certificate at age 18/university), income (split into quartiles from Q1—lowest to Q4—highest), home ownership (owns outright/owns with mortgage/rents or other), and internet use, classified as infrequent/never (once a year or less, several times a year, several times a month) or frequent (several times a week, every day or almost every day). Internet use was included as an indicator of digital inequality, reflecting differential access to information, services, and online social engagement, which is not captured by more universal behaviors such as telephone use or meeting friends and family. Participants’ home postcodes were used to determine, for their geographical area, the level of deprivation based on the index of multiple deprivation (IMD) and rurality (urban/rural). Additional potential sources of inequalities are described in the Supplementary Material.

#### Outcomes

Quality of life was assessed with the Quality of Life in Alzheimer’s Disease scale (Logsdon et al., 2000). This includes 13 questions asking respondents to rate aspects of life covering physical health, energy level, mood, living situation, memory, relationships with family, relationships with spouse/partner, relationships with friends, self-perception, ability to do things for fun, financial situation, overall life evaluation, and ability to complete everyday tasks. Items are rated on a 4-point scale (poor, fair, good, or excellent). Total scores range from 13 to 52, with higher scores indicating better quality of life. Satisfaction with Life Scale (Diener et al., 1985) is a 5-item scale on which respondents rate their satisfaction with aspects of life on a 7-point Likert-type scale. Items assess whether respondents perceive their life as close to their ideal, whether their life conditions are excellent, overall life satisfaction, whether they have obtained important life goals, and whether they would change almost nothing if they could live their life over. Possible scores range from 5 to 35, with higher scores reflecting greater satisfaction. World Health Organization-Five Well-Being Index (Bech, 2004) is a 5-item scale on which respondents rate their level of psychological well-being in relation to mood, activity, and interests using a six-point Likert-type scale. Items assess whether respondents have felt cheerful, calm and relaxed, active and vigourous, and fresh and rested after sleep, as well as whether their daily life has been filled with things that interest them. Possible scores range from 0 to 25, with higher scores indicating greater well-being; scores were transformed to a percentage scale (0–100).

#### Statistical analyses

**Figure 1:**
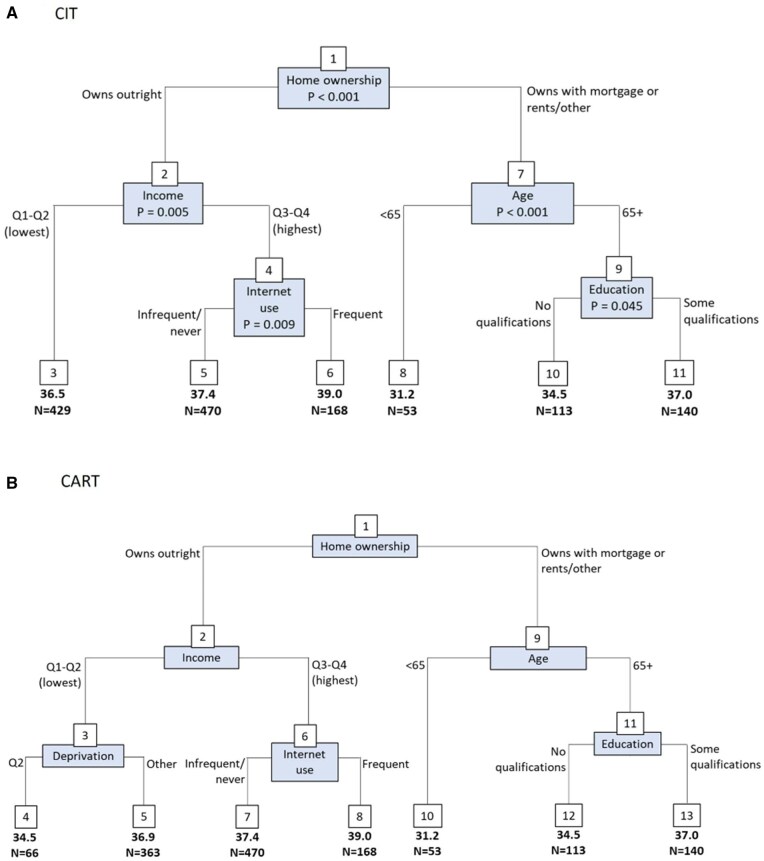
Regression trees for measures of inequality and quality of life (*n *= 1,373). The numbers in boxes are the node numbers. The nodes at the bottom of the tree are the terminal nodes, and the mean quality of life scores are reported for each terminal node. Cross-validated *R*^2^: (A) 0.042, (B) 0.041.

Descriptive statistics were reported as the number and percentage of people in each category for each study measure. Mean scores on the outcome measures were reported alongside standard deviations. Regression analysis exploring the relationship between the study measures and quality of life, satisfaction with life, or well-being is described in the Supplementary ­Material (Supplementary Tables 1–3 and Supplementary ­Figure 1).

The standard approach for intersectional analysis is the multilevel analysis of individual heterogeneity and discriminatory analysis (MAIHDA). This involves constructing a full intersectional stratum, which in this case would involve cross-classifying all social positions of the above-mentioned social dimensions: 5(age group) × 2(sex) × 2(ethnicity) × 2(marital status) × 2(living situation) × 4(education) × 3(occupation) × 4(income) × 2(home ownership) × 2(internet use) × 5(deprivation quintiles) × 2(urban/rural). This creates 153,600 possible combinations. However, many of the strata were empty of observations, with 418 intersectional strata containing at least one observation (averaged across 25 imputations to account for missing data). Using the full intersectional matrix representing all possible combinations of each measure of inequality leads to methodological limitations such as cell sizes that are too small and insufficient power for operationalization, is computationally challenging, and results in findings that are difficult to interpret. We therefore took the approach of reducing the complexity of the intersectional interactions by using regression trees to identify intersectional strata as previously described (Pedrós Barnils et al., 2025).

The goal of the regression tree is to create a reduced intersectional matrix that captures the key social dimensions. This approach is exploratory and designed to identify empirically homogenous subgroups. Regression trees are a quantitative, non-parametric, exploratory form of recursive partitioning in which the sample population is divided into subgroups based on the factors of interest and can support the detection of subgroups with higher or lower levels of quality of life, satisfaction with life, or well-being. The root node at the top of the tree contains the whole sample, and the sample is split based on the splitting rule of the tree, which aims to maximize the homogeneity of the outcome within subgroups. This is done recursively at each sub-node until the stopping criteria are met. More details are provided in the Supplementary Material.

For each of the three outcomes (quality of life, satisfaction with life, and well-being), two regression trees were built: a Classification and Regression Tree (CART) and a Conditional Inference Tree (CIT). Only participants who had a score on the given outcome measure were included in the analyses. Additional information on these models is provided in the Supplementary Material, including full settings for each tree. As the trees use different models and splitting specifications, we compared results from the two models to check the robustness of findings. The CART was built using the *rpart* package in R, and the CIT and random forest were built using the *partykit* package. To keep the results interpretable, the depth of the trees was limited to three. For both trees, relaxation of the splitting criteria was explored to check for over-fitting. Because regression trees can be vulnerable to random patterns in the data, a random forest of 500 trees was used to determine the variable importance measure of each measure of inequality, indicating whether the splitting variable also had the highest importance across an ensemble of trees (Strobl et al., 2009). Additional details are found in the Supplementary Material.

To check that it was possible to identify sources of inequality beyond the effects of co-morbidity and type of dementia, which are known to influence “living well,” we first ran the trees for quality of life, including these two variables. To check that age was not masking the effect of other measures of inequality, we conducted a sensitivity analysis for each of the three outcome measures with age removed.

#### The reduced intersectional strata

The reduced intersectional strata were extracted from the terminal nodes of the CIT regression trees. A linear regression employed the reduced intersectional strata as the main exposure (categorical variable with the highest score on the outcome as the reference category), which was used to estimate the effect sizes of the associations with outcomes.

#### Interaction terms among top splitters

To corroborate the findings from the trees, linear models with interaction terms amongst the top splitters were conducted, and marginal means were reported.

## Results

### Sample characteristics

The sample included 1,537 people with dementia. More than half were male, and nearly 40% were aged 80 or above. Over 50% had a diagnosis of Alzheimer’s disease, and almost 50% had two or more co-morbid conditions. Most of the sample were of white ethnicity. Three-quarters were married or cohabiting, and 19% lived alone. Regarding socio-economic position, 42% had higher managerial, administrative, or professional occupational status, over 50% had been educated to age 18 or longer, while 29% had no educational qualifications, and over three-quarters (77%) owned their homes outright. Technology use was relatively limited, with only 24% using the internet frequently. Two-thirds lived in urban areas, and over 50% were in the two least deprived IMD quintiles. Table 1 reports, for each factor of interest, the number of people in each category for the total sample and for respondents with scores on each of the three living well measures (quality of life, satisfaction with life, and well-being), together with mean scores for those measures.

**Table 1:** Descriptive statistics for study measures and scores on quality of life, satisfaction with life, and well-being.

Study measure	Response	Total sample (*N *= 1,537)	QoL-AD (*n *= 1,373)		SwLS (*n *= 1,494)		WHO-5 (*n *= 1,511)	
		*n* (%)	*n* (%)	Mean (*SD*)	*n* (%)	Mean (*SD*)	*n* (%)	Mean (*SD*)
***Personal characteristics***								
**Age group**	<65	134 (8.7)	121 (8.8)	34.9 (6.6)	133 (8.9)	22.9 (7.0)	133 (8.8)	57.3 (24.9)
	65–69	177 (11.5)	161 (11.7)	36.3 (6.6)	176 (11.8)	25.5 (6.4)	176 (11.6)	57.6 (21.8)
	70–74	258 (16.8)	230 (16.8)	36.8 (5.8)	249 (16.7)	25.8 (5.9)	252 (16.7)	59.5 (20.7)
	75–79	366 (23.8)	326 (23.7)	37.1 (5.9)	354 (23.7)	26.7 (5.7)	358 (23.7)	61.1 (20.2)
	80+	602 (39.2)	535 (39.0)	37.2 (5.5)	582 (39.0)	26.8 (5.8)	592 (39.2)	63.3 (18.9)
**Sex**	Male	865 (56.3)	779 (56.7)	36.7 (6.0)	841 (56.3)	26.1 (6.0)	851 (56.3)	61.6 (20.2)
	Female	672 (43.7)	594 (43.3)	36.9 (5.8)	653 (43.7)	26.0 (6.2)	660 (43.7)	60.2 (21.0)
**Type of dementia**	AD	851 (55.4)	766 (55.8)	37.7 (5.5)	829 (55.5)	26.9 (5.7)	840 (55.6)	63.9 (19.5)
	VaD	170 (11.1)	149 (10.9)	35.3 (6.6)	162 (10.8)	24.9 (6.7)	163 (10.8)	56.3 (21.7)
	Mixed AD and VaD	324 (21.1)	294 (21.4)	36.3 (5.8)	315 (21.1)	25.9 (6.1)	317 (21.0)	59.3 (20.6)
	FTD	54 (3.5)	48 (3.5)	38.6 (5.6)	52 (3.5)	25.7 (5.9)	53 (3.5)	62.9 (20.6)
	PDD	44 (2.9)	39 (2.8)	33.0 (5.6)	44 (3.0)	22.2 (6.7)	44 (2.9)	48.1 (20.2)
	LBD	53 (3.4)	44 (3.2)	32.8 (6.3)	52 (3.5)	22.5 (6.2)	53 (3.5)	49.5 (18.5)
	Other/unspecified	41 (2.7)	33 (2.4)	34.9 (7.8)	40 (2.7)	26.1 (7.2)	41 (2.7)	59.1 (24.8)
**Ethnicity^a^**	White	1,492 (98.9)	1,340 (98.8)	36.8 (5.9)	1,453 (98.8)	26.1 (6.1)	1,472 (98.9)	60.9 (20.1)
	Other	17 (1.1)	16 (1.2)	34.9 (6.9)	17 (1.2)	25.6 (6.6)	16 (1.1)	56.3 (25.3)
	Missing	28	17	–	24	–	23	–
**Marital status^b^**	Married	1,153 (75.0)	1,043 (76.0)	37.0 (6.0)	1,125 (75.3)	26.5 (6.0)	1,132 (75.0)	61.6 (20.6)
	Other	384 (25.0)	330 (24.0)	36.3 (5.7)	369 (24.7)	24.8 (6.2)	379 (25.0)	58.2 (20.3)
**Lives alone/with others**	Living with others	1,247 (81.2)	1,124 (82.0)	36.9 (6.0)	1,215 (81.4)	26.6 (6.0)	1,225 (81.2)	61.6 (20.6)
	Living alone	288 (18.8)	247 (18.0)	36.3 (5.6)	277 (18.6)	24.0 (6.2)	284 (18.8)	58.2 (20.3)
	Missing	2	2	–	2	–	2	–
**Number of co-morbid conditions**	0	334 (22.7)	294 (22.3)	38.8 (5.8)	322 (22.5)	26.8 (5.7)	329 (22.7)	66.9 (19.4)
	1	421 (28.6)	387 (29.3)	37.4 (5.4)	415 (28.9)	26.6 (5.7)	419 (28.9)	62.4 (18.8)
	2+	717 (48.7)	638 (48.4)	35.6 (6.0)	697 (48.6)	25.6 (6.4)	701 (48.4)	57.5 (21.2)
	Missing	65	54	–	60	–	62	–
***Socioeconomic position***								
**Education**	No qualifications	429 (29.0)	386 (28.2)	35.9 (6.0)	415 (27.9)	25.9 (6.3)	424 (28.2)	59.2 (21.2)
	School leaving certificate at 16	272 (17.8)	240 (17.5)	37.1 (5.7)	267 (17.9)	26.0 (6.2)	269 (17.9)	61.4 (21.0)
	School leaving certificate at 18	519 (33.9)	481 (35.2)	37.1 (5.8)	504 (33.8)	26.4 (5.7)	509 (33.8)	61.4 (20.1)
	University	311 (20.3)	261 (19.1)	37.4 (6.0)	304 (20.4)	25.9 (6.2)	303 (20.1)	62.2 (20.0)
	Missing	6	5	–	4	–	6	–
**Occupation**	1 Higher managerial, administrative, and professional occupations	631 (41.9)	564 (41.9)	37.2 (5.8)	612 (41.8)	26.1 (6.1)	619 (41.8)	62.2 (20.0)
	2 Intermediate occupations	448 (29.7)	398 (29.6)	37.1 (6.0)	437 (29.8)	26.1 (5.9)	442 (29.8)	61.6 (20.2)
	3 Routine and manual occupations	428 (28.4)	383 (28.5)	36.0 (5.8)	416 (28.4)	26.1 (6.2)	421 (28.4)	58.9 (21.2)
	Missing	30	28	–	29	–	29	–
**Income quartiles**	Q1 Lowest income	277 (27.3)	249 (26.9)	35.5 (5.8)	266 (26.8)	25.0 (6.2)	273 (27.3)	58.0 (20.8)
	Q2	279 (27.5)	251 (27.2)	36.5 (6.1)	274 (27.6)	26.4 (6.0)	275 (27.5)	61.2 (21.8)
	Q3	250 (24.6)	228 (24.7)	37.6 (5.9)	243 (24.5)	27.2 (5.4)	246 (24.6)	63.4 (19.4)
	Q4 Highest income	210 (20.7)	196 (21.2)	38.1 (5.6)	208 (21.0)	26.8 (5.9)	207 (20.7)	63.5 (20.3)
	Missing	521	449	–	503	–	510	–
**Home ownership**	Owns outright	1,181 (76.8)	1,067 (77.7)	37.3 (5.7)	1,150 (77.0)	26.5 (5.9)	1,164 (77.0)	62.1 (19.8)
	Owns with mortgage	89 (6.1)	77 (4.8)	35.7 (7.2)	87 (6.1)	24.7 (7.3)	89 (6.2)	59.1 (25.4)
	Rents/other	262 (17.0)	226 (16.5)	34.8 (6.1)	253 (23.6)	24.8 (6.4)	254 (16.8)	56.0 (21.1)
	Missing	5	3	–	4	–	4	–
**Internet use**	Infrequent or never	1,156 (75.7)	1,036 (75.8)	36.7 (5.9)	1,117 (75.1)	26.3 (5.9)	1,136 (75.5)	61.2 (20.5)
	Frequent	372 (24.3)	330 (24.2)	37.1 (6.0)	371 (24.9)	25.5 (6.5)	369 (24.5)	60.4 (20.8)
	Missing	9	7	–	6	–	6	–
***Area-level characteristics***								
**Deprivation**	Q1 most deprived	124 (8.1)	113 (8.2)	34.9 (5.8)	120 (8.0)	24.7 (7.0)	121 (8.0)	59.0 (20.7)
	Q2	232 (15.1)	207 (15.1)	35.6 (5.9)	225 (15.1)	25.8 (6.0)	230 (15.2)	57.2 (22.2)
	Q3	335 (21.8)	295 (21.5)	36.8 (5.7)	328 (22.0)	25.9 (6.0)	329 (21.8)	60.9 (21.1)
	Q4	382 (24.9)	343 (25.0)	37.2 (6.2)	371 (24.8)	26.0 (6.2)	377 (25.0)	61.5 (20.3)
	Q5 least deprived	464 (30.2)	415 (30.2)	37.6 (5.7)	450 (30.1)	26.8 (5.8)	454 (30.0)	63.0 (19.2)
**Urban/rural**	Urban	1,033 (67.2)	933 (68.0)	36.8 (6.0)	1,006 (67.3)	26.2 (6.0)	1,016 (67.2)	60.7 (20.6)
	Rural	504 (32.8)	440 (32.1)	36.9 (5.7)	488 (32.7)	25.9 (6.3)	495 (32.8)	61.5 (20.5)

*Note*. QoL-AD = Quality of Life in Alzheimer’s Disease Scale; SwLS = Satisfaction with Life Scale; WHO-5 = World Health Organization-Five Well-Being Index; *SD* = standard deviation; AD = Alzheimer’s disease; VaD = vascular dementia; FTD = frontotemporal dementia; PDD = Parkinson’s disease dementia; LBD = dementia with Lewy bodies.

a Ethnicity was classified as White (White British/White other) or other (Bangladeshi, Indian, Pakistani, Black-African or Caribbean, mixed).

b Marital status was classified as married (married/has a partner/is cohabiting) or other (single/divorced/separated/widowed).

### Identification of reduced intersectional strata with homogenous quality of life scores

There were 1,373 people with dementia who had a score for quality of life. We first ran the trees, including the number of co-morbid conditions and the type of dementia. While these two factors provided the strongest differentiation, home ownership and age also emerged as key factors across the two trees (Supplementary Figure 2). We therefore proceeded to run the trees excluding co-morbidity and type of dementia.

As shown in Figure 1A and B, CIT and CART grew almost identical trees to three levels. CIT produced 6 distinct subgroups incorporating 5 of the 12 measures of inequality while the CART tree produced a further subgroup split by deprivation. In both cases, owning the home outright (vs owning with a mortgage, or rents/other) was the strongest factor associated with differences in quality of life.

Those who owned their home outright were differentiated by income, with those in the two highest income quartiles further differentiated by internet use. As shown in Figure 1, for both CIT and CART trees, those who had the highest quality of life owned their home outright, had the highest income, and used the internet frequently (CIT node 6/CART node 8; mean = 39.0, *N *= 168). For those who owned their home outright, those in the bottom two income quartiles (node 3 for CIT; mean = 36.5, *N *= 429) had the lowest quality of life scores. This node split further by deprivation in the CART tree, with the lowest quality of life seen for those who were in IMD quintile 2 (node 4; mean = 34.5, *N *= 66).

Among those who did not own their home outright (either owns with a mortgage, or rents/other), those aged <65 (CIT node 8/CART node 10; mean = 31.2, *N *= 53) had the lowest quality of life scores. For those aged 65+, those with some educational qualifications had higher quality of life scores than those with no qualifications. To check that age was not masking the effect of other measures of inequality, sensitivity analysis was conducted with age removed. As shown in Supplementary Figure 3, the composition of the measures within the trees remained similar when age was removed.

A random forest was used to validate the measures in the trees. All the measures identified as being most relevant in the trees were also the most important measures as determined by the random forest (Supplementary Figure 4).

The six intersectional strata identified from the CIT tree are described in Table 2. Linear regression shows that the people with dementia who own their home outright, have higher levels of income, and use the internet have the highest quality of life, with the mean score approximately 8 points (percentage-of-scale change: 20%) higher than the mean score for those with the lowest quality of life who fall in the strata of owns with mortgage/rents/other under the age of 65.

**Table 2:** Reduced intersectional strata identified from the CIT trees for quality of life, satisfaction with life, and well-being.

Intersectional strata	Node	*N*	Mean score	Estimate (95% CI)
**Quality of life (ranked from low to high QoL-AD scores)**				
** 1 Home rented or mortgaged, aged <65**	8	53	31.2	−8.04 (−9.85, −6.23)
** 2 Home rented or mortgaged, aged 65+, no qualifications**	10	113	34.5	−4.68 (−6.10, −3.26)
** 3 Owns home outright, lower income**	3	429	36.5	−2.61 (−3.70, −1.53)
** 4 Home rented or mortgaged, aged 65+, some qualifications**	11	140	37.0	−2.23 (−3.57, −0.88)
** 5 Owns home outright, higher income, non-internet user**	5	470	37.4	−1.67 (−2.81, −0.53)
** 6 Owns home outright, higher income, internet user**	6	168	39.0	Ref
**Satisfaction with life (ranked from low to high SwLS scores)**				
** 1 Lives alone, aged <75**	3	64	21.7	−5.65 (−7.16, −4.14)
** 2 Lives with others, aged <65**	7	110	23.8	−3.55 (−4.74, −2.37)
** 3 Lives alone, aged 75+**	4	215	24.8	−2.54 (−3.44, −1.64)
** 4 Lives with others, aged 65–74**	8	384	26.0	−1.34 (−2.07, −0.61)
** 5 Lives with others, aged 75+**	9	721	27.3	Ref
**Well-being (ranked from low to high WHO-5 scores)**				
** 1 Does not own home outright or with mortgage, aged <75**	4	103	48.9	−13.01 (−17.09, −8.93)
** 2 Does not own home outright or with mortgage, aged 75+**	5	151	60.8	−1.10 (−4.52, 2.33)
** 3 Owns home outright or with mortgage**	2	1,257	62.0	Ref

*Note*. QoL-AD = Quality of Life in Alzheimer’s Disease Scale; SwLS = Satisfaction with Life Scale; WHO-5 = World Health Organization-Five Well-Being Index; CI = confidence interval; Ref = reference category.

To validate the patterns identified by the decision tree, we modelled interactions between the key splitting variables (home ownership, age, income). The corresponding marginal means, shown in Supplementary Table 4, are consistent with the tree-based results.

### Identification of reduced intersectional strata with homogenous satisfaction with life scores

**Figure 2:**
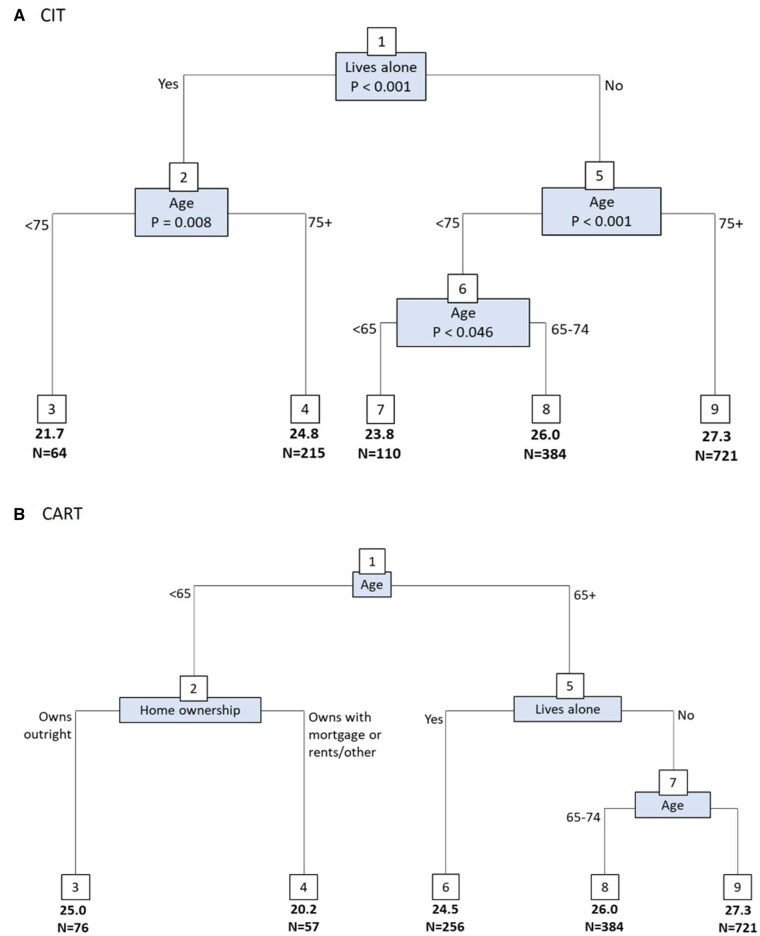
Regression trees for measures of inequality and satisfaction with life (*n *= 1,494). The numbers in boxes are the node numbers. The nodes at the bottom of the tree are the terminal nodes, and the mean satisfaction with life scores are reported for each terminal node. Cross-validated *R*^2^: (A) 0.036, (B) 0.030.

There were 1,494 people with dementia with a score for satisfaction with life. The composition of the CIT and CART trees differed for satisfaction with life (Figure 2), although the most salient factors appeared in both trees. For the CIT tree, living alone provided the strongest differentiation, followed by age. People who lived alone and were aged <75 (CIT node 3; mean = 21.7, *n *= 64) had the lowest scores for satisfaction with life. Among people who did not live alone, there was further differentiation by age. Those aged 75+ who lived with others had the highest satisfaction with life scores (CIT node 9; mean 27.3, *n *= 721). Those aged <65 had the lowest satisfaction with life scores of those who lived with others (CIT node 7; mean 23.8, *n* = 110). In the CART tree, home ownership further differentiated satisfaction with life scores, with those aged <65 who do not own their home outright having the lowest satisfaction with life scores (CART node 4; mean 20.2, *n *= 57).

**Figure 3:**
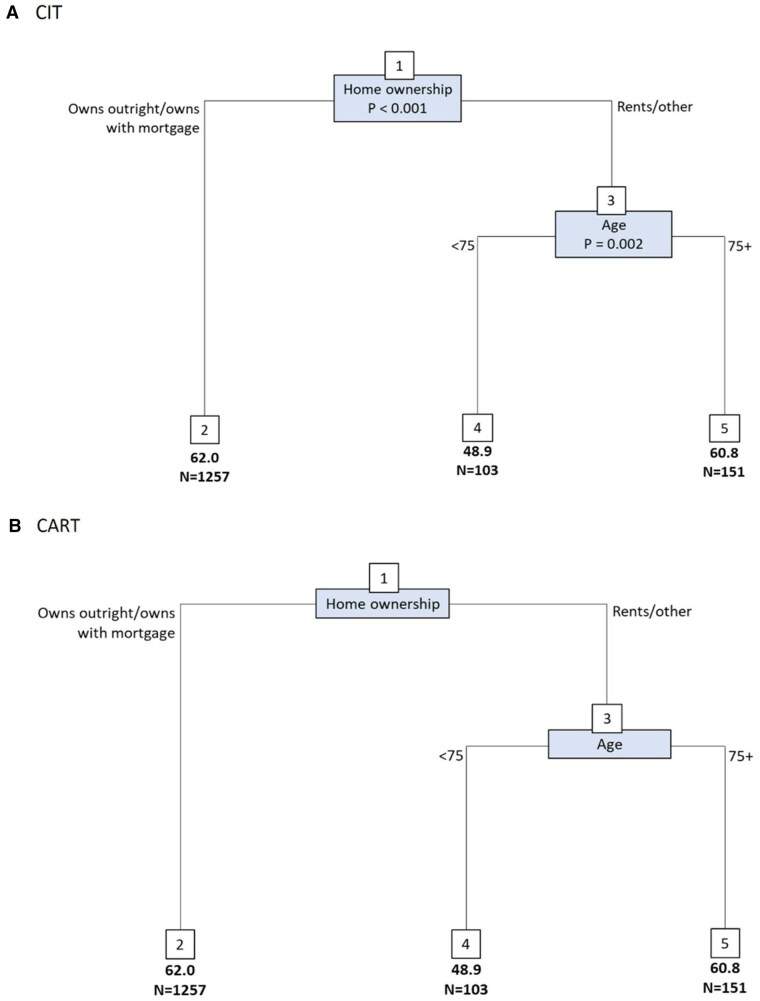
Regression trees for measures of inequality and well-being (*n *= 1,511). The numbers in boxes are the node numbers. The nodes at the bottom of the tree are the terminal nodes, and the mean well-being scores are reported for each terminal node. Cross-validated *R*^2^: (A) 0.008, (B) 0.009.

When age was removed from the models (Supplementary Figure 5), the CIT and CART trees were identical. Living situation was the strongest differentiating factor. Those living alone had the lowest scores for satisfaction with life (node 2; mean = 24.1, *n *= 279), while those who lived with others and owned their home outright had the highest scores (node 4; mean = 26.9, *n *= 972).

The random forest used to validate the importance of the measures in the trees can be found in Supplementary Figure 6. The measures identified as being most relevant in the trees were amongst the most important measures as determined by the random forest.

The reduced intersectional matrix, based on the CIT tree, comprised five groups described in Table 2. Linear regression revealed that people with dementia who live with others and are aged 75+ have the highest satisfaction with life scores, which is approximately 6 points (percentage-of-scale change: 19%) higher than those with the lowest satisfaction with life score who fall into the strata that live alone and are under the age of 75.

To validate the patterns identified in the decision tree, we modeled interactions between the key splitting variables (living situation and age). The corresponding marginal means, shown in Supplementary Table 5, are consistent with the tree-based results.

### Identification of reduced intersectional strata with homogenous well-being scores

There were 1,511 people with dementia with a score for well-being. The CIT and CART trees produced similar findings for well-being (Figure 3). For both, home ownership was the strongest differentiating factor. For well-being, homeowners (own outright or with a mortgage) grouped together. Those who were not homeowners (rent/other) were further differentiated by age, with the lowest well-being seen among those who did not own their home and were aged <75 (node 4; mean = 48.9, *n *= 103). The highest well-being scores were seen in those who owned their own home (node 2; mean 62.0, *n *= 1,257). Relaxing the α splitting criterion for the CIT tree further differentiated this group according to living situation; those living with others had higher well-being scores than those who lived alone (mean = 62.9, *n *= 981). When age was removed, the models showed the same composition (Supplementary Figure 7).

The random forest used to validate the measures in the trees can be found in Supplementary Figure 8. The measures identified as being most relevant in the trees were also the most important measures as determined by the random forest.

The reduced intersectional matrix comprised three groups described in Table 2. Linear regression revealed that people with dementia who own their own home (either outright or with a mortgage) have the highest well-being scores, approximately 13 points (percentage-of-scale change: 13%) higher than those with the lowest well-being scores, who fall into the strata that do not own their own home and are under the age of 75.

To validate the patterns identified in the decision tree, we modelled interactions between the key splitting variables (home ownership and age). The corresponding marginal means, shown in Supplementary Table 6, are consistent with the tree-based results.

## Discussion and implications

Using data from the British IDEAL cohort, this secondary analysis applied regression-tree analysis for the first time to explore how the intersection of factors beyond differences associated with type of dementia and degree of co-morbidity can support detection of subgroups of people with dementia experiencing higher or lower levels of quality of life, satisfaction with life, and well-being, indicating capability for “living well” with the condition. Exploration of intersectionality using quantitative methods is in its early stages, and whilst MAIHDA is the gold standard, it requires large sample sizes and relatively few study measures. Regression trees are an approach to overcoming these challenges, allowing data-driven exploration of heterogeneity within populations based on social identities and positions. Factors considered reflected personal characteristics (age and living situation), socioeconomic position (home ownership, income, educational qualifications, and internet use), and geographical location (area-level deprivation and rurality). Age, living situation, and home ownership emerged as the strongest differentiating factors. Those people with dementia who fared best in terms of “living well” were older homeowners with educational qualifications and relatively higher incomes who lived with others. Those who fared less well were younger, did not own their own home, had no educational qualifications, and/or lived alone. Geographical location was less salient. These findings shed light on how combinations of factors contribute to inequalities in the capability to “live well” among community-dwelling people with mild-to-moderate dementia. In particular, and drawing on the concept of precarity, interconnections between inequalities in personal and societal resources and a range of individual vulnerabilities can lead to the accumulation of pressures and shape individuals’ lived experiences.

Cumulative pressures arise from aspects of people’s personal characteristics and living situations. Younger people with dementia often face a prolonged wait for a correct diagnosis, and the diagnosis often comes at a difficult stage of family life, where people are still paying mortgages, have children at home, and look after older parents. Carers often reduce their working hours to provide care, with significant financial implications. There is a lack of suitable health and social care services as well as a lack of companionship and activity options, leaving people with many unmet needs (Carter et al., 2018). People with dementia living alone are more vulnerable simply because there is no one immediately available if needed, but while many have good support from family and friends, others have little or no support, putting them especially at risk of harm (Charles et al., 2015). The proportion of older people, and consequently also people with dementia, living alone is growing, but there are fewer long-term services and little support tailored to the needs of this group, who are predominantly older women on low incomes (Clare et al., 2024) and are more likely to be admitted to residential care (Chen et al., 2014).

Cumulative pressures equally arise from structural inequalities linked to socio-economic position. These have been most extensively explored in relation to the risk of developing dementia. Limited education, low income, not being a homeowner, and not using the internet are among the psychosocial and environmental stressors that increase risk (Cho et al., 2023; Lai et al., 2022). These factors continue to be relevant once dementia develops and are linked with greater barriers to accessing services. For example, people on low incomes are diagnosed with dementia later than more affluent individuals and are less likely to access early support and intervention (Petersen et al., 2021). Home ownership is an important indicator of socioeconomic position in British society and is associated with better health overall (Munford et al., 2020). Tenants with dementia may find themselves in properties that are not well-maintained and potentially harmful to health, struggle to afford the rent, worry about the insecurity of tenure, or be at risk of eviction. Previous analysis of IDEAL cohort data showed that, while individual socio-economic position was more impactful, area-level deprivation was also relevant as a source of inequality (Wu et al., 2018); it is associated with poorer access to services and poorer quality care (Watson et al., 2023). In the present analysis, area-level deprivation was relevant only for quality of life, and we did not have an explanation as to why people in Quintile 2 fared worst. Similar to earlier analyses (Wu et al., 2018), no differences were found between urban and rural populations.

While the IDEAL cohort was reasonably diverse in respect of personal characteristics, socio-economic position, and geographical location, there were some significant limitations. As a secondary analysis, the study was necessarily limited to variables collected in the original IDEAL baseline assessment, restricting the range of factors that could be investigated. Variation in item-level missingness meant that analytic samples differed slightly across the three living well measures, although these differences were small. The use of regression-tree models also introduces methodological considerations, as tree structures can be sensitive to small variations and do not provide effect-size estimates. The sample was 99% white, reflecting the current age distribution of older people in Britain, where relatively few people from minority ethnic communities have yet reached the ages at which dementia is most common. This limited the potential for considering inequalities associated with ethnicity. The present study could therefore miss important differences between ethnic groups and underestimate inequalities that may exist across different communities. Research from the United Kingdom indicates that people from ethnic minority backgrounds experience structural and cultural barriers to dementia diagnosis and care, including language or stigma-related barriers, underdiagnosis, and less access to culturally appropriate support (Kenning et al., 2017). Similarly, other potential sources of inequity that would have been of interest, such as those associated with identifying as LGBTQ+, could not be explored due to small numbers. Nevertheless, the IDEAL cohort is the largest study of people living with dementia in Great Britain, with a well-characterized sample that reasonably reflects the characteristics of the population attending memory services in the United Kingdom (Prince et al., 2014). The findings presented here demonstrate how multiple factors contribute to inequalities and are associated with “living well” with dementia, providing a basis for further exploration of the intersection of a wider range of possible sources of inequality.

A focus on “living well,” although useful in raising expectations, can mask the fact that receiving a diagnosis of dementia and living with the condition involves grief, loss, and psychological pain, and indeed suffering (Bartlett et al., 2017). Our findings suggest it may also mask the extent to which the structures, systems, and material resources needed to make “living well” possible are unequally distributed. Socio-economic and geographical disparities contribute to disadvantage and are exacerbated for our sample by the retrenchment in the public sector in the United Kingdom since the adoption of “austerity” as a cornerstone of policy following the global financial crisis of 2008–2009 (Hernandez, 2021). This disproportionately affects those people with dementia who are already more prone to precarity, through reduced access to health and social care services, preventive services, and social housing, and greater threats to personal safety, among other disadvantages. This is the situation in the United Kingdom, an advanced economy with relatively well-developed health care services; globally, disparities are much greater and the associated challenges probably more extensive (Leist, 2017).

Addressing the challenge of dementia requires more than timely diagnosis and symptom management, especially at a time when resources are increasingly channeled to new, still unproven, expensive treatments for the minority (Walsh, 2023), risking further reductions in resources allocated to care for the majority. Socio-economic and geographical disparities in the potential for “living well” with the condition can only be mitigated through policy and system change. Achieving equity may require greater focus on groups and areas that are less well served, attention to specific barriers, including cultural relevance and accessibility, and flexibility in approach, for example, working through community organizations (Ekezie et al., 2023). The World Health Organization Global Action Plan (World Health Organization, 2017) puts forward a vision of “a world where people with dementia live well and receive the care and support they need to fulfil their potential with dignity, respect, autonomy and equality” (pp. 4–5), based on underpinning principles including equity, universal health and social care coverage, and multisector collaboration. Yet action has been slow and key targets remain unmet; at the time of writing, only 48 countries (i.e., one-third of the target) have a dementia plan in place. Social inequalities mean that many people with dementia do not receive optimal care. Addressing this requires concerted effort and robust policy responses (Leist, 2017).

### Conclusions

We began by asking whether there are observable disparities among groups of people with dementia in the capability to ‘live well’ and whether such disparities amount to inequities by nature of being avoidable, unnecessary, or unjust. Building on analyses of UK data indicating the relevance of discrete factors with an intersectional approach, and drawing on the concept of precarity, findings show that socio-economic and geographical disparities are associated with the capability to ‘live well’ with the condition. For those less advantaged, the effects of these disparities intersect and combine to result in the cumulative pressures that characterize precarity and reduce the potential for “living well”; many people with dementia are not receiving the right care and support. These findings from a cohort of people who have already accessed services and received a dementia diagnosis in an advanced Western economy may represent only a fraction of the broader national and especially global challenges. Addressing the impact of these social inequalities requires a sustained focus on developing and implementing policies that help to reduce disparities and create conditions in which people with dementia can “live well” and flourish.

## Supplementary Material

igag009_Supplementary_Data

## Data Availability

This study involved secondary analysis of data from the IDEAL cohort. IDEAL data were deposited with the UK data archive in April 2020. Details of how the data can be accessed are available at: https://reshare.ukdataservice.ac.uk/854293/.
