# Supplementary material for: Sources of inequality in “living well” with dementia: an intersectional analysis using a British cohort study
Source: Innov Aging. 2026 Jan 29;10(4):igag009. doi: 10.1093/geroni/igag009 (PMC13049594; doi:10.1093/geroni/igag009)
Supplement: igag009_Supplementary_Data [file igag009_supplementary_data.docx]

***Innovation in Aging* Supplementary Material: Clare, Gamble, Martyr, Knapp, & Matthews. Sources of inequality in ‘living well’ with dementia: an intersectional analysis using a British cohort study****.**

**Supplementary Methods**

**Control variables for regression analysis**

We included time elapsed since diagnosis (<1 year, 1-2 years, 3-5 years, 6+ years), and cognition to check that observed differences were not attributable simply to length of time spent living with dementia or severity of dementia symptoms. Cognition was assessed with the Addenbrooke’s Cognitive Examination-III (ACE-III). Scores range from 0-100 with higher scores reflecting better cognition (Hsieh et al., 2013). Age and sex were also controlled for, except where age or sex were the factor of interest.

**Statistical analyses**

***Regression***

Only participants who had a score on the relevant outcome were included in the analyses. First, the association between each factor of interest and the outcome was evaluated using linear regression, adjusting for age group, sex, dementia diagnosis, and time elapsed since diagnosis (Model 1). Age and sex were treated as baseline confounders, given their potential to influence both socioeconomic position and living well, and were included in all models. Type of dementia and time since diagnosis were also included in all models as clinical adjustment factors. Although these variables may be influenced, at least in part, by socioeconomic circumstances (e.g., via access to diagnostic services or differential pathways to diagnosis), they also capture meaningful clinical heterogeneity that could otherwise obscure associations of interest. For models in which age group or sex was the exposure, the remaining covariates were included. Factors that showed evidence of an association with the outcome were then entered together in a multivariable model to assess whether any were independently associated with the outcome when considered simultaneously (Model 2). Models including ACE-III are presented as sensitivity analyses only (Models 3 and 4). These models are used to evaluate the robustness of observed associations when accounting for cognitive differences, which may lie downstream of socioeconomic circumstances. Models 1 and 2 were repeated with the addition of ACE-III scores (Models 3 and 4). Throughout, we interpret the coefficients as associations, not causal effects, and do not claim that adjustment sets isolate direct causal pathways. See Supplementary Figure 1. To account for missing data, multiple imputation was carried out generating 25 imputed datasets. Estimates were combined according to Rubin’s rules. Regression analysis was conducted in Stata 16.

***Regression trees – additional information***

A minimum number of 50 observations per node is recommended to retain statistical robustness (Harper, 2005; Lemon et al., 2003). Regression-tree methods intrinsically handle missing values in the study measures. In the first step, observations with missing values on the splitting variable are ignored. The observations with missing values are then assigned to child nodes by so-called surrogate splits, which mimic the decision rule as closely as possible (Strobl et al., 2009).

In CART (Breiman, 1984), a regression tree is used for numerical outcomes, and the splits are not based on statistical significance but aim to minimise the variance. CART methods are prone to overfitting, and to develop a tree of the best size and lowest misclassification rate, the tree is pruned based on the complexity parameter associated with the minimum cross-validation error (Venkatasubramaniam et al., 2017). In CIT (Hothorn et al., 2006), the selection and splitting process are separated, and the splitting variables are determined using statistical significance (α-level of 5% in this case, with Bonferroni correction for multiple testing). In the first step, the variable with the strongest bivariate association with the outcome is selected, and in the second step the algorithm identifies the optimal binary partition based on the study measure. Whereas CART has a bias towards selecting variables with many possible splits or missing values, CIT enables an unbiased selection procedure. For both trees, the algorithm terminates when the stopping criteria are met. For CIT, the algorithm also terminates if none of the study measures show a significant association with the outcome at the specified α-level.

***Tree settings***

*CIT settings: ctree* uses permutation test for conditional independence at each node. For numeric responses, this is a linear statistic (correlation-based test). The threshold for splitting (mincriterion) is 0.95 which corresponds to α = 0.05 after Bonferroni correction. The minimum number of observations (minsplit) in a node before it is considered for splitting = 20. The minimum number of observations in a terminal node (minbucket) = 50, and the maximum depth of the tree (maxdepth) = 3.

*CART settings:* fitted *using rpart with* the default complexity parameter (cp = 0.01). The minimum split size = 20, the minimum terminal node size = 50, and the maximum depth of the tree = 3. *Rpart* performs 10-fold cross-validation to estimate the prediction error for different tree sizes. The final tree was selected using the complexity parameter corresponding to the minimum cross-validated error.

***Random Forest for variable importance***

Variable importance was assessed using a random forest regression model implemented in the *partykit* package (functions cforest and varimp). Importance was computed as the increase in out-of-bag mean squared error (MSE) under conditional permutation, which is appropriate for continuous outcomes. Random forests were fit with default settings: trees grown on bootstrap samples with replacement, and the number of predictors considered at each split (mtry) set to ⌊√p⌋. Importance values were computed within each imputed dataset and averaged across imputations to obtain final estimates.

**Supplementary Results**

**Associations of the factors of interest with quality of life**

Linear regressions were conducted to assess the cross-sectional associations of the factors of interest with quality of life (Supplementary Table 1). Initially, the association of each individual factor with quality of life was assessed (Model 1). There was evidence that a younger age, not being married and living alone (vs living with others) were associated with poorer quality of life. Having no qualifications, a more manual occupation, a lower income, not owning a home outright and infrequent internet use were associated with poorer quality of life, as was residing in a more deprived area. There was no evidence of an association between sex, ethnicity or living in an urban or rural location and quality of life. The measures of inequality found to be important for quality of life in Model 1 were entered into a multivariable model to determine whether any were associated with quality of life independently of the other measures (Model 2). A lower age, not owning a home outright and living in a more deprived area remained independently associated with poorer quality of life. To determine whether these findings were associated with cognitive function, the models were repeated with adjustment for cognition (ACE-III) (Models 3 and 4). Findings remained similar, except that internet use became less important.

**Associations of the factors of interest with satisfaction with life**

There were 1494 people with dementia who had a score for satisfaction with life. Linear regressions were conducted to assess the associations between each measure of inequality and satisfaction with life (Supplementary Table 2). Similar to quality of life, there was evidence that a younger age, not being married, living alone, a lower income, not owning a home outright, and living in a more deprived area were associated with poorer satisfaction with life. Sex, ethnicity, living in an urban or rural location, internet use, and educational and occupational status were not associated with satisfaction with life. Living alone and age were found to remain independently associated with satisfaction with life in the multivariable model and this remained following adjustment for cognition.

**Associations of the factors of interest with well-being**

There were 1511 people with dementia who had a score for well-being. Linear regressions were conducted to assess the associations between each measure of inequality and well-being (Supplementary Table 3). Similar to quality of life, there was evidence that a younger age, not being married, living alone, having no qualifications, having a lower income, not owning a home outright, and living in a more deprived area were associated with poorer well-being. Additionally, being female was associated with lower well-being. Age, living alone, and deprivation remained independently associated with well-being in the multivariable model and this remained following adjustment for cognition.

**Supplementary Table 1.** Association of potential sources of inequality with quality of life

| **Quality of Life** | **Model 1.**  **Univariable.**  **Adjusted for age, sex, diagnosis, and time elapsed since diagnosis** | **Model 2.**  **Multivariable.**  **Adjusted for age, sex, diagnosis, and time elapsed since diagnosis** | **Model 3.**  **Univariable.**  **Adjusted for age, sex, diagnosis, time elapsed since diagnosis, ACE-III** | **Model 4.**  **Multivariable.**  **Adjusted for age, sex, diagnosis, time elapsed since diagnosis, ACE-III** |
| --- | --- | --- | --- | --- |
|  | **Estimate (95% CI)** | **Estimate (95% CI)** | **Estimate (95% CI)** | **Estimate (95% CI)** |
| *Personal characteristics* | | | | |
| Age group |  |  |  |  |
| <65 | -3.04 (-4.20, -1.88) | -2.82 (-4.05, -1.59) | -3.06 (-4.22, -1.90) | -2.63 (-3.82, -1.43) |
| 65-69 | -1.14 (-2.16, -0.11) | -1.30 (-2.38, -0.22) | -1.29 (-2.32, -0.26) | -1.24 (-2.29, -0.19) |
| 70-74 | -0.42 (-1.31, 0.47) | -0.65 (-1.56, 0.26) | -0.51 (-1.40, 0.38) | -0.63 (-1.53, 0.30) |
| 75-79 | -0.10 (-0.89, 0.70) | -0.31 (-1.11, 0.50) | -0.12 (-0.91, 0.67) | -0.28 (-1.08, 0.52) |
| 80+ | Ref | Ref | Ref | Ref |
| Sex |  |  |  |  |
| Male | Ref | Ref | Ref | Ref |
| Female | -0.01 (-0.63, 0.61) | 0.19 (-0.47, 0.85) | 0.10 (-0.52, 0.73) | 0.23 (-0.43, 0.89) |
| Ethnicity^a^ |  |  |  |  |
| White | Ref | - | Ref | - |
| Other | -1.71 (-4.55, 1.12) | - | -1.20 (-4.06, 1.65) | - |
| Marital status^b^ |  |  |  |  |
| Married | Ref | Ref | Ref | Ref |
| Other | -0.90 (-1.67, -0.13) | -0.04 (-1.29, 1.21) | -0.96 (-1.73, -0.20) | -0.03 (-1.28, 1.22) |
| Lives alone/with others |  |  |  |  |
| Living with others | Ref | Ref | Ref | Ref |
| Living alone | -0.81 (-1.63, 0.00) | -0.06 (-1.40, 1.28) | -0.89 (-1.73, -0.05) | -0.12 (-1.46, 1.23) |
| *Socio-economic position* | | | | |
| Education |  |  |  |  |
| No qualifications | Ref | Ref | Ref | Ref |
| School leaving certificate at 16 | 1.41 (0.48, 2.33) | 0.88 (-0.07, 1.83) | 1.33 (0.40, 2.26) | 0.85 (-0.11, 1.80) |
| School leaving certificate at 18 | 1.41 (0.62, 2.19) | 0.77 (-0.08, 1.61) | 1.29 (0.50, 2.09) | 0.72 (-0.12, 1.57) |
| University | 1.55 (0.64, 2.47) | 0.55 (-0.55, 1.65) | 1.36 (0.42, 2.29) | 0.50 (-0.59, 1.59) |
| Occupation |  |  |  |  |
| 1 Higher managerial, administrative and professional occupations | Ref | Ref | Ref | Ref |
| 2 Intermediate occupations | -0.08 (-0.83, 0.67) | 0.44 (-0.38, 1.25) | -0.02 (-0.77, 0.74) | 0.45 (-0.36, 1.27) |
| 3 Routine and manual occupations | -1.08 (-1.84, -0.32) | 0.05 (-0.83, 0.93) | -0.94 (-1.71, -0.17) | 0.08 (-0.80, 0.95) |
| Income quartiles |  |  |  |  |
| Q1 Lowest income | Ref | Ref | Ref | Ref |
| Q2 | 0.74 (-0.22, 1.70) | 0.52 (-0.41, 1.45) | 0.68 (-0.28, 1.65) | 0.33 (-0.66, 1.33) |
| Q3 | 1.63 (0.66, 2.60) | 1.03 (-0.06, 2.13) | 1.58 (0.62, 2.54) | 1.05 (0.01, 2.09) |
| Q4 Highest income | 2.07 (1.06, 3.07) | 1.22 (0.03, 2.35) | 2.03 (1.02, 3.03) | 1.36 (0.21, 2.52) |
| Home ownership |  |  |  |  |
| Owns outright | Ref | Ref | Ref | Ref |
| Owns with mortgage | -0.73 (-2.08, 0.63) | -0.36 (-1.70, 0.98) | -0.77 (-2.08, 0.53) | -0.41 (-1.70, 0.88) |
| Rents/other | -2.18 (-3.00, -1.36) | -1.09 (-1.99, -0.19) | -1.88 (-2.67, -1.09) | -0.89 (-1.76, -0.02) |
| Internet use |  |  |  |  |
| Infrequent or never | Ref | Ref | Ref | - |
| Frequent | 0.88 (0.12, 1.63) | 0.53 (-0.24, 1.30) | 0.61 (-0.19, 1.40) | - |
| *Area-level characteristics* | | | | |
| Deprivation |  |  |  |  |
| Q1 most deprived | -2.38 (-3.59, -1.17) | -1.12 (-2.40, 0.16) | -2.31 (-3.51, -1.10) | -1.13 (-2.41, 0.15) |
| Q2 | -1.85 (-2.81, -0.80) | -1.24 (-2.23, -0.26) | -1.78 (-2.74- -0.83) | -1.19 (-2.18, -0.21) |
| Q3 | -0.62 (-1.48, 0.23) | -0.21 (-1.08, 0.66) | -0.62 (-1.47, 0.23) | -0.23 (-1.10, 0.64) |
| Q4 | -0.33 (-1.15, 0.49) | -0.06 (-0.89, 0.76) | -0.34 (-1.16, 0.48) | -0.09 (-0.92, 0.73) |
| Q5 least deprived | Ref | Ref | Ref | Ref |
| Urban/rural |  |  |  |  |
| Urban | Ref | - | Ref | - |
| Rural | 0.09 (-0.57, 0.74) | - | 0.06 (-0.60, 0.71) | - |

*Note*. CI, confidence intervals; ACE-III, Addenbrooke’s Cognitive Examination-III. Models 1 and 3 are univariable in that they have only one study measure in the model, but they are adjusted for covariates. Multivariable models include multiple study measures in addition to covariates. Note that age and sex are both study variables and covariates (they are adjusted for in all models, except where they are the variable of interest).

^a^ Ethnicity was classified as white (white British/white other) or other (Bangladeshi, Indian, Pakistani, black-African or Caribbean, mixed)

^b^ Marital status was classified as married (married/has a partner/is cohabiting) or other (single/divorced/separated/widowed).

**Supplementary Table 2.** Association of potential sources of inequality with satisfaction with life

| **Satisfaction with life** | **Model 1.**  **Univariable.**  **Adjusted for age, sex, diagnosis, and time elapsed since diagnosis** | **Model 2.**  **Multivariable.**  **Adjusted for age, sex, diagnosis, and time elapsed since diagnosis** | **Model 3.**  **Univariable.**  **Adjusted for age, sex, diagnosis, time elapsed since diagnosis, ACE-III** | **Model 4.**  **Multivariable.**  **Adjusted for age, sex, diagnosis, time elapsed since diagnosis, ACE-III** |  |
| --- | --- | --- | --- | --- | --- |
|  | **Estimate (95% CI)** | **Estimate (95% CI)** | **Estimate (95% CI)** | **Estimate (95% CI)** |  |
| *Personal characteristics* | | | | | |
| Age group |  |  |  |  |  |
| <65 | -4.19 (-5.33, -3.05) | -4.16 (-5.32, -2.99) | -4.19 (-5.33, -3.05) | -4.16 (-5.33, -3.00) |  |
| 65-69 | -1.30 (-2.31, -0.30) | -1.72 (-2.74, -0.70) | -1.30 (-2.31, -0.29) | -1.74 (-2.76, -0.71) |  |
| 70-74 | -0.90 (-1.78, -0.03) | -1.35 (-2.24, -0.47) | -0.90 (-1.78, -0.02) | -1.36 (-2.25, -0.47) |  |
| 75-79 | -0.06 (-0.84, 0.71) | -0.55 (-1.33, 0.24) | -0.06 (-0.84, 0.72) | -0.55 (-1.34, 0.24) |  |
| 80+ | Ref | Ref | Ref | Ref |  |
| Sex |  |  |  |  |  |
| Male | Ref | Ref | Ref | Ref |  |
| Female | -0.45 (-1.05, 0.16) | 0.10 (-0.55, 0.74) | -0.45 (-1.06, 0.16) | 0.10 (-0.54, 0.75) |  |
| Ethnicity^a^ |  |  |  |  |  |
| White | Ref | - | Ref | - |  |
| Other | -0.12 (-2.93, 2.70) | - | -0.14 (-2.98, 2.71) | - |  |
| Marital Status^b^ |  |  |  |  |  |
| Married | Ref | Ref | Ref | Ref |  |
| Other | -2.13 (-2.87, -1.39) | -0.36 (-0.87, 1.58) | -2.14 (-2.88, -1.39) | -0.36 (-0.87, 1.59) |  |
| Lives alone/with others |  |  |  |  |  |
| Living with others | Ref | Ref | Ref | Ref |  |
| Living alone | -2.92 (-3.73, -2.12) | -2.83 (-4.14, -1.52) | -2.94 (-3.75, -2.14) | -2.84 (-4.16, -1.52) |  |
| *Socio-economic position* | | | | | |
| Education |  |  |  |  |  |
| No qualifications | Ref | Ref | Ref | Ref |  |
| School leaving certificate at 16 | 0.55 (-0.37, 1.46) | 0.24 (-0.67, 1.15) | 0.56 (-0.36, 1.47) | 0.23 (-0.68, 1.15) |  |
| School leaving certificate at 18 | 0.77 (-0.02, 1.56) | 0.53 (-0.28, 1.33) | 0.78 (-0.01, 1.58) | 0.51 (-0.30, 1.32) |  |
| University | 0.27 (-0.62, 1.16) | -0.16 (-1.11, 0.79) | 0.29 (-0.62, 1.19) | -0.18 (-1.15, 0.78) |  |
| Occupation |  |  |  |  |  |
| 1 Higher managerial, administrative and professional occupations | Ref | - | Ref | - |  |
| 2 Intermediate occupations | 0.19 (-0.54, 0.93) | - | 0.19 (-0.55, 0.93) | - |  |
| 3 Routine and manual occupations | 0.18 (-0.56, 0.93) | - | 0.18 (-0.57, 0.94) | - |  |
| Income quartiles |  |  |  |  |  |
| Q1 Lowest income | Ref | Ref | Ref | Ref |  |
| Q2 | 1.05 (0.13, 1.97) | 0.51 (-0.44, 1.45) | 1.06 (0.14, 1.98) | 0.50 (-0.45, 1.45) |  |
| Q3 | 1.72 (0.75, 2.69) | 0.85 (-0.18, 1.88) | 1.73 (0.75, 2.70) | 0.85 (-0.18, 1.88) |  |
| Q4 Highest income | 1.52 (0.54, 2.49) | 0.58 (-0.55, 1.71) | 1.52 (0.54, 2.50) | 0.58 (-0.56, 1.71) |  |
| Home ownership |  |  |  |  |  |
| Owns outright | Ref | Ref | Ref | Ref |  |
| Owns with mortgage | -0.94 (-12.25, 0.38) | -0.86 (-2.17, 0.45) | -0.93 (-2.23, 0.38) | -0.93 (-2.22, 0.36) |  |
| Rents/other | -1.36 (-2.17, -0.56) | -0.43 (-1.31, 0.46) | -1.21 (-2.01, -0.42) | -0.30 (-1.18, 0.57) |  |
| Internet use |  |  |  |  |  |
| Infrequent or never | Ref | - | Ref | - |  |
| Frequent | -0.33 (-1.06, 0.41) | - | -0.35 (-1.13, 0.43) | - |  |
| *Area-level characteristics* | | | | | |
| Deprivation |  |  |  |  |  |
| Q1 most deprived | -1.36 (-3.57, -1.16) | -0.52 (-1.78, 0.74) | -1.37 (-3.58, -1.17) | -0.52 (-1.78, 0.75) |  |
| Q2 | -0.74 (-1.68, 0.21) | -0.30 (-1.26, 0.66) | -0.74 (-1.69, 0.21) | -0.30 (-1.26, 0.67) |  |
| Q3 | -0.57 (-1.41, 0.27) | -0.26 (-1.11, 0.59) | -0.57 (-1.41, 0.27) | -0.26 (-1.11, 0.59) |  |
| Q4 | -0.44 (-1.25, 0.38) | -0.26 (-1.07, 0.55) | -0.44 (-1.25, 0.38) | -0.26 (-1.07, 0.55) |  |
| Q5 least deprived | Ref | Ref | Ref | Ref |  |
| Urban/rural |  |  |  |  |  |
| Urban | Ref | - | Ref | - |  |
| Rural | -0.34 (-0.98, 0.30) | - | -0.34 (-0.98, 0.31) | - |  |

*Note*. CI, confidence intervals; ACE-III, Addenbrooke’s Cognitive Examination-III. Models 1 and 3 are univariable in that they have only one study measure in the model, but they are adjusted for covariates. Multivariable models include multiple study measures in addition to covariates. Note that age and sex are both study variables and covariates (they are adjusted for in all models, except where they are the variable of interest).

^a^ Ethnicity was classified as white (white British/white other) or other (Bangladeshi, Indian, Pakistani, black-African or Caribbean, mixed)

^b^ Marital status was classified as married (married/has a partner/is cohabiting) or other (single/divorced/separated/widowed).

**Supplementary Table 3.** Association of potential sources of inequality with well-being

| **Well-being** | **Model 1.**  **Univariable.**  **Adjusted for age, sex, diagnosis, and time elapsed since diagnosis** | **Model 2.**  **Multivariable.**  **Adjusted for age, sex, diagnosis, and time elapsed since diagnosis** | **Model 3.**  **Univariable.**  **Adjusted for age, sex, diagnosis, time elapsed since diagnosis, ACE-III** | **Model 4.**  **Multivariable.**  **Adjusted for age, sex, diagnosis, time elapsed since diagnosis, ACE-III** |  |
| --- | --- | --- | --- | --- | --- |
|  | **Estimate (95% CI)** | **Estimate (95% CI)** | **Estimate (95% CI)** | **Estimate (95% CI)** |  |
| *Personal characteristics* | | | | | |
| Age group |  |  |  |  |  |
| <65 | -7.79 (-11.67, -3.91) | -7.07 (-11.20, -2.93) | -7.70 (-11.58, -3.82) | -7.02 (-11.04, -3.00) |  |
| 65-69 | -6.20 (-9.61, -2.79) | -6.23 (-9.86, -2.60) | -5.95 (-9.39, -2.52) | -5.97 (-9.50, -2.44) |  |
| 70-74 | -3.92 (-6.89, -0.95) | -4.49 (-7.55, -1.43) | -3.77 (-6.74, -0.80) | -4.33 (-7.37, -1.30) |  |
| 75-79 | -2.25 (-4.89, 0.38) | -2.64 (-5.34, 0.07) | -2.19 (-4.82, 0.45) | -2.53 (-5.22, 0.16) |  |
| 80+ | Ref | Ref | Ref | Ref |  |
| Sex |  |  |  |  |  |
| Male | Ref | Ref | Ref | Ref |  |
| Female | -2.35 (-4.41, -0.29) | -1.89 (-4.10, 0.33) | -2.51 (-4.58, -0.43) | -2.04 (-4.26, 0.18) |  |
| Ethnicity^a^ |  |  |  |  |  |
| White | Ref | - | Ref | - |  |
| Other | -3.99 (-13.92, 5.95) | - | -4.99 (-15.02, 5.05) | - |  |
| Marital Status^b^ |  |  |  |  |  |
| Married | Ref | Ref | Ref | Ref |  |
| Other | -2.66 (-5.18, -0.13) | -2.75 (-1.40, 6.89) | -2.57 (-5.10, -0.04) | -2.70 (-1.44, 6.84) |  |
| Lives alone/with others |  |  |  |  |  |
| Living with others | Ref | Ref | Ref | Ref |  |
| Living alone | -4.08 (-6.82, -1.33) | -4.08 (-6.82, -1.33) | -3.96 (-6.71, -1.20) | -4.49 (-6.90, -0.09) |  |
| *Socio-economic position* | | | | | |
| Education |  |  |  |  |  |
| No qualifications | Ref | Ref | Ref | Ref |  |
| School leaving certificate at 16 | 3.10 (0.02, 6.18) | 2.05 (-1.12, 5.23) | 3.30 (0.22, 6.39) | 2.19 (-0.99, 5.37) |  |
| School leaving certificate at 18 | 2.36 (-0.29, 5.01) | 1.18 (-1.69, 4.04) | 2.66 (-0.01, 5.32) | 1.37 (-1.49, 4.24) |  |
| University | 3.18 (0.17, 6.20) | 1.51 (-2.12, 5.14) | 3.65 (0.59, 6.71) | 1.81 (-1.81, 5.43) |  |
| Occupation |  |  |  |  |  |
| 1 Higher managerial, administrative and professional occupations | Ref | Ref | Ref | Ref |  |
| 2 Intermediate occupations | -0.11 (-2.60, 2.38) | 0.98 (-1.78, 3.73) | -0.27 (-2.77, 2.22) | 0.90 (-1.85, 3.66) |  |
| 3 Routine and manual occupations | -2.52 (-5.05, 0.01) | -0.28 (-3.27, 2.72) | -2.82 (-5.38, -0.27) | -0.44 (-3.43, 2.55) |  |
| Income quartiles |  |  |  |  |  |
| Q1 Lowest income | Ref | Ref | Ref | Ref |  |
| Q2 | 2.29 (-0.75, 5.33) | 1.16 (-2.03, 4.36) | 2.39 (-0.65, 5.44) | 1.26 (-1.94, 4.45) |  |
| Q3 | 4.67 (1.55, 7.79) | 3.06 (-0.41, 6.53) | 4.78 (1.65, 7.91) | 3.13 (-0.35, 6.61) |  |
| Q4 Highest income | 4.53 (1.32, 7.73) | 2.61 (-1.18, 6.40) | 4.62 (1.42, 7.82) | 2.61 (-1.16, 6.38) |  |
| Home ownership |  |  |  |  |  |
| Owns outright | Ref | Ref | Ref | Ref |  |
| Owns with mortgage | -1.25 (-5.67, 3.17) | -0.44 (-4.84, 3.96) | -1.07 (-5.37, 3.23) | -0.38 (-4.65, 3.90) |  |
| Rents/other | -5.33 (-8.05, -2.61) | -2.93 (-5.94, 0.08) | -4.58 (-7.23, -1.93) | -2.32 (-5.25, 0.61) |  |
| Internet use |  |  |  |  |  |
| Infrequent or never | Ref | - | Ref | - |  |
| Frequent | 0.28 (-2.21, 2.78) | - | 0.86 (-1.75, 3.48) | - |  |
| *Area-level characteristics* | | | | | |
| Deprivation |  |  |  |  |  |
| Q1 most deprived | -3.00 (-7.08, 1.08) | 0.07 (-4.27, 4.41) | -3.14 (-7.23, 0.94) | 0.01 (-4.33, 4.34) |  |
| Q2 | -5.01 (-8.20, -1.82) | -3.62 (-6.92, -0.32) | -5.17 (-8.26, -1.97) | -3.72 (-7.02, -0.42) |  |
| Q3 | -1.25 (-4.11, 1.60) | -0.34 (-3.25, 2.57) | -1.28 (-4.13, 1.57) | -0.33 (-3.24, 2.58) |  |
| Q4 | -0.62 (-3.37, 2.13) | -0.19 (-2.96, 2.57) | -0.62 (-3.37, 2.12) | -0.18 (-2.94, 2.59) |  |
| Q5 least deprived | Ref | Ref | Ref | Ref |  |
| Urban/rural |  |  |  |  |  |
| Urban | Ref | - | Ref | - |  |
| Rural | -0.79 (-1.37, 2.96) | - | -0.85 (-1.32, 3.01) | - |  |

*Note*. CI, confidence intervals; ACE-III, Addenbrooke’s Cognitive Examination-III. Models 1 and 3 are univariable in that they have only one study measure in the model, but they are adjusted for covariates. Multivariable models include multiple study measures in addition to covariates. Note that age and sex are both study variables and covariates (they are adjusted for in all models, except where they are the variable of interest).

^a^ Ethnicity was classified as white (white British/white other) or other (Bangladeshi, Indian, Pakistani, black-African or Caribbean, mixed)

^b^ Marital status was classified as married (married/has a partner/is cohabiting) or other (single/divorced/separated/widowed).

**Supplementary Table 4.** Interactions amongst top splitters for QoL: Home ownership, age, and income

| **Variables** | **Marginal mean (95% CI)** |
| --- | --- |
| Owns home outright | 37.2 (36.9, 37.6) |
| Owns home with mortgage | 37.0 (34.9, 39.0) |
| Rents/other | 35.3 (34.2, 36.4) |
|  |  |
| Age |  |
| <65 | 36.4 (35.2, 37.5) |
| 65-69 | 36.2 (35.3, 37.1) |
| 70-74 | 36.7 (35.9, 37.4) |
| 75-79 | 37.0 (36.4, 37.6) |
| 80+ | 37.3 (36.8, 37.9) |
|  |  |
| Owns home outright x age <65 | 37.7 (36.4, 39.1) |
| Owns home outright x age 65-69 | 36.7 (35.7, 37.8) |
| Owns home outright x age 70-74 | 36.8 (36.0, 37.7) |
| Owns home outright x age 75-79 | 37.2 (36.5, 37.9) |
| Owns home outright x age 80+ | 37.4 (36.9, 37.9) |
|  |  |
| Owns home with mortgage x age <65 | 32.1 (29.4, 34.7) |
| Owns home with mortgage x age 65-69 | 36.1 (32.9, 39.2) |
| Owns home with mortgage x age 70-74 | 35.8 (33.0, 38.6) |
| Owns home with mortgage x age 75-79 | 37.6 (34.8, 40.4) |
| Owns home with mortgage x age 80+ | 38.5 (34.0, 42.9) |
|  |  |
| Rents/other x age <65 | 31.0 (28.9, 33.1) |
| Rents/other x age 65-69 | 33.5 (31.3, 35.8) |
| Rents/other x age 70-74 | 35.9 (33.7, 38.2) |
| Rents/other x age 75-79 | 35.6 (33.6, 37.5) |
| Rents/other x age 80+ | 36.4 (35.0, 27.8) |
|  |  |
| Income Q1 | 35.7 (35.0, 36.4) |
| Income Q2 | 36.6 (35.9, 37.3) |
| Income Q3 | 37.4 (36.7, 38.1) |
| Income Q4 | 37.5 (36.7, 38.4) |
|  |  |
| Owns home outright x Income Q1 | 36.2 (35.3, 37.0) |
| Owns home outright x Income Q2 | 36.9 (36.1, 37.6) |
| Owns home outright x Income Q3 | 37.7 (37.0, 38.4) |
| Owns home outright x Income Q4 | 38.4 (37.6, 39.1) |
|  |  |
| Owns home with mortgage x Income Q1 | 35.4 (30.9, 39.9) |
| Owns home with mortgage x Income Q2 | 36.5 (33.7, 39.3) |
| Owns home with mortgage x Income Q3 | 38.7 (35.9, 41.6) |
| Owns home with mortgage x Income Q4 | 37.6 (34.0, 41.2) |
|  |  |
| Rents/other x Income Q1 | 34.5 (33.4, 35.5) |
| Rents/other x Income Q2 | 36.0 (34.3, 37.7) |
| Rents/other x Income Q3 | 36.3 (34.0, 38.7) |
| Rents/other x Income Q4 | 34.4 (30.6, 38.2) |

**Supplementary Table 5.** Interactions amongst top splitters for SwLS: Lives alone, age, and home ownership

| **Variables** | **Marginal mean (95% CI)** |
| --- | --- |
| Lives alone | 36.3 (35.5, 37.1) |
| Lives with others | 36.8 (36.5, 37.2) |
|  |  |
| Age |  |
| <65 | 36.5 (35.3, 37.6) |
| 65-69 | 36.1 (35.2, 37.1) |
| 70-74 | 36.5 (35.7, 37.3) |
| 75-79 | 37.0 (36.3, 37.6) |
| 80+ | 37.1 (36.6, 37.6) |
|  |  |
| Lives alone x age <65 | 35.2 (32.6, 37.8) |
| Lives alone x age 65-69 | 35.2 (31.8, 38.6) |
| Lives alone x age 70-74 | 35.9 (33.5, 38.3) |
| Lives alone x age 75-79 | 37.3 (35.5, 39.1) |
| Lives alone x age 80+ | 36.6 (35.7, 37.5) |
|  |  |
| Lives with others x age <65 | 36.7 (35.5, 38.0) |
| Lives with others x age 65-69 | 36.3 (35.4, 37.3) |
| Lives with others x age 70-74 | 36.6 (35.8, 37.4) |
| Lives with others x age 75-79 | 36.9 (36.2, 37.6) |
| Lives with others x age 80+ | 37.2 (36.6, 37.8) |
|  |  |
| Owns home outright | 37.3 (36.9, 37.6) |
| Owns home with mortgage | 36.1 (34.4, 37.9) |
| Rents/other | 34.9 (34.2, 35.7) |
|  |  |
| Owns home outright x age <65 | 38.0 (36.6, 39.4) |
| Owns home outright x age 65-69 | 36.8 (35.6, 38.0) |
| Owns home outright x age 70-74 | 36.9 (36.0, 37.8) |
| Owns home outright x age 75-79 | 37.3 (36.6, 38.1) |
| Owns home outright x age 80+ | 37.3 (36.8, 37.9) |
|  |  |
| Owns home with mortgage x age <65 | 32.6 (30.0, 35.2) |
| Owns home with mortgage x age 65-69 | 34.9 (32.0, 37.7) |
| Owns home with mortgage x age 70-74 | 36.3 (33.7, 39.0) |
| Owns home with mortgage x age 75-79 | 37.3 (34.3, 40.2) |
| Owns home with mortgage x age 80+ | 36.6 (32.7, 40.5) |
|  |  |
| Rents/other x age <65 | 30.8 (28.8, 32.9) |
| Rents/other x age 65-69 | 33.6 (31.5, 35.7) |
| Rents/other x age 70-74 | 34.7 (32.6, 36.7) |
| Rents/other x age 75-79 | 35.2 (33.4, 37.0) |
| Rents/other x age 80+ | 36.3 (35.1, 37.5) |

**Supplementary Table 6.** Interactions amongst top splitters for WHO-5: Home ownership and age

| **Variables** | **Marginal mean (95% CI)** |
| --- | --- |
|  |  |
| Owns home outright | 62.2 (61.1, 63.4) |
| Owns home with mortgage | 61.5 (56.0, 67.0) |
| Rents/other | 56.5 (53.9, 59.0) |
|  |  |
| Age |  |
| <65 | 61.0 (57.2, 64.7) |
| 65-69 | 57.7 (54.7, 60.8) |
| 70-74 | 59.2 (56.7, 61.7) |
| 75-79 | 60.9 (58.8, 63.0) |
| 80+ | 63.3 (61.6, 65.0) |
|  |  |
| Owns home outright x age <65 | 65.2 (60.6, 69.8) |
| Owns home outright x age 65-69 | 59.5 (55.9, 63.0) |
| Owns home outright x age 70-74 | 60.6 (57.7, 63.4) |
| Owns home outright x age 75-79 | 61.3 (59.0, 63.6) |
| Owns home outright x age 80+ | 63.7 (61.8, 65.5) |
|  |  |
| Owns home with mortgage x age <65 | 49.7 (41.1, 58.4) |
| Owns home with mortgage x age 65-69 | 59.3 (50.0, 68.7) |
| Owns home with mortgage x age 70-74 | 61.4 (52.5, 70.3) |
| Owns home with mortgage x age 75-79 | 65.0 (55.9, 74.1) |
| Owns home with mortgage x age 80+ | 62.8 (50.8, 74.7) |
|  |  |
| Rents/other x age <65 | 45.7 (39.2, 52.3) |
| Rents/other x age 65-69 | 49.3 (42.3, 56.3) |
| Rents/other x age 70-74 | 52.1 (45.3, 58.9) |
| Rents/other x age 75-79 | 57.8 (51.7, 63.9) |
| Rents/other x age 80+ | 62.0 (58.2, 65.8) |


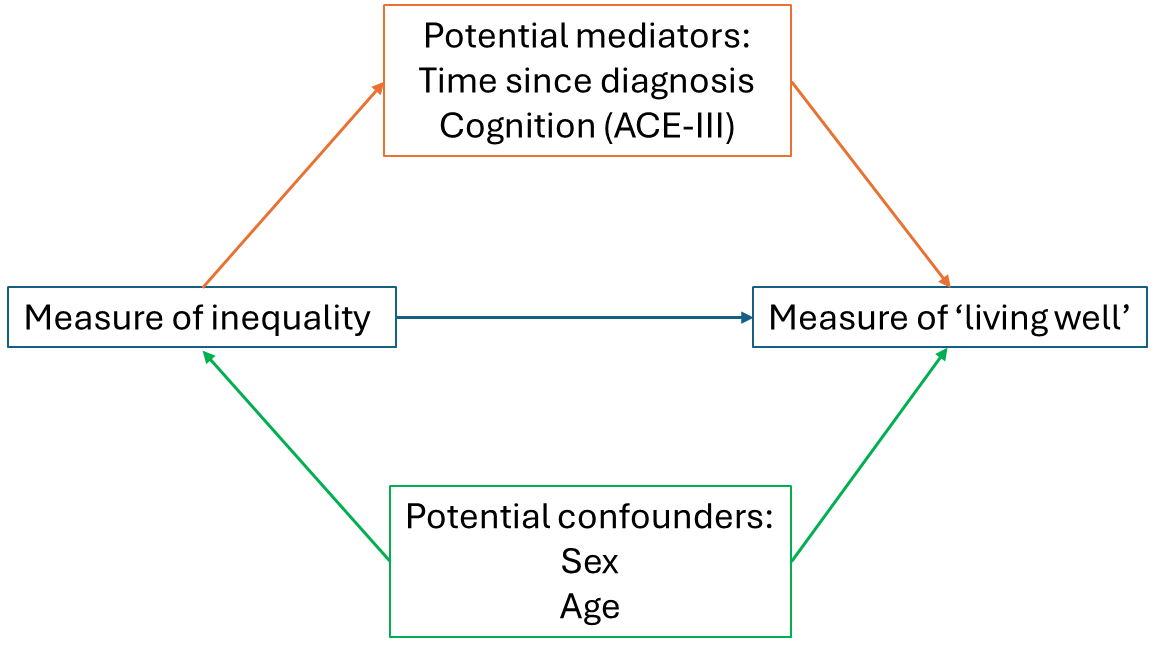


**Supplementary Figure 1.** Conceptual diagram illustrating assumed relationships between socioeconomic position (measure of inequality), clinical variables, and measures of “living well” (quality of life, satisfaction with life, and well-being).
Age and sex are treated as confounders. Diagnosis and time since diagnosis, though potentially influenced by socioeconomic position, are included in all models as clinical adjustment factors to account for relevant disease heterogeneity. Along with time since diagnosis, cognitive functioning (ACE-III) is considered a potential mediator between socioeconomic position and quality of life and ACE-III is therefore included only in sensitivity analyses. All model estimates are interpreted as associations rather than causal effects.

**Supplementary Figure 2.** Regression trees for quality of life and measures of inequality including number of co-morbidities and type of dementia

1. CIT


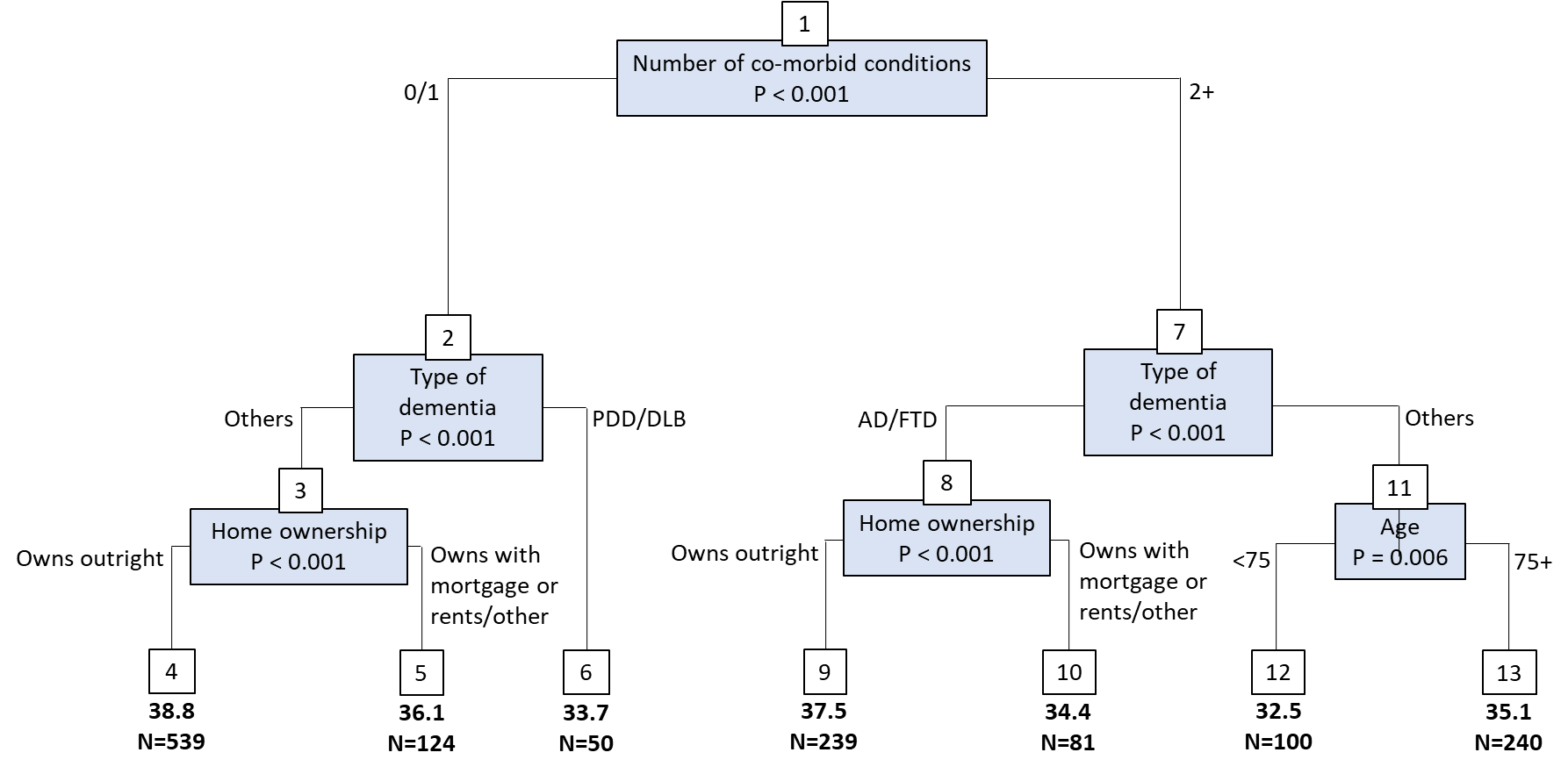


1. CART


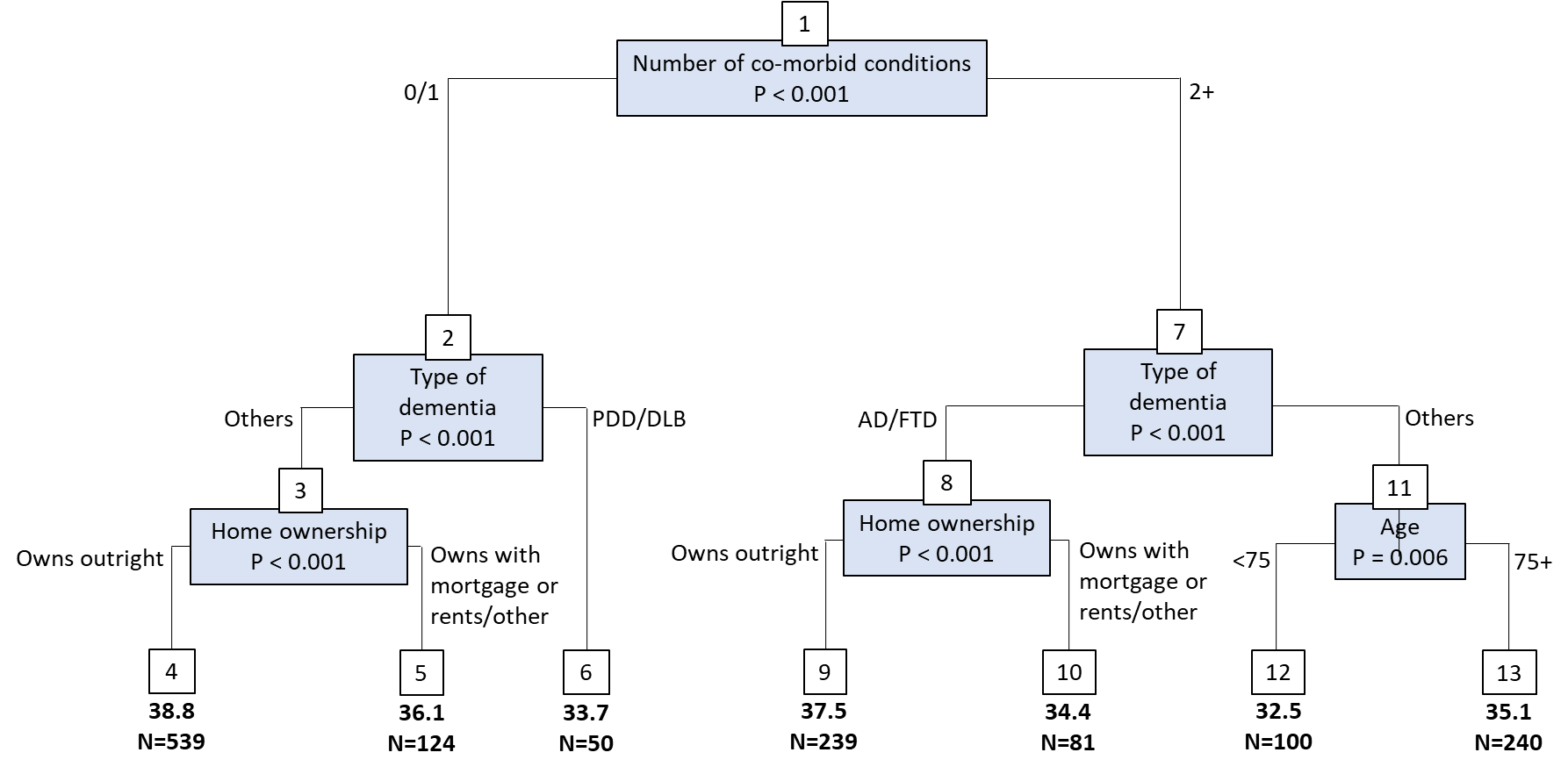


*Note*. The numbers in boxes are the node numbers. The nodes at the bottom of the tree are the terminal nodes, and the mean quality of life scores are reported for each terminal node. Cross-validated R^2^: A) 0.065, B) 0.062.

**Supplementary Figure 3.** Regression trees for quality of life and measures of inequality with age removed from the analysis

1. CIT


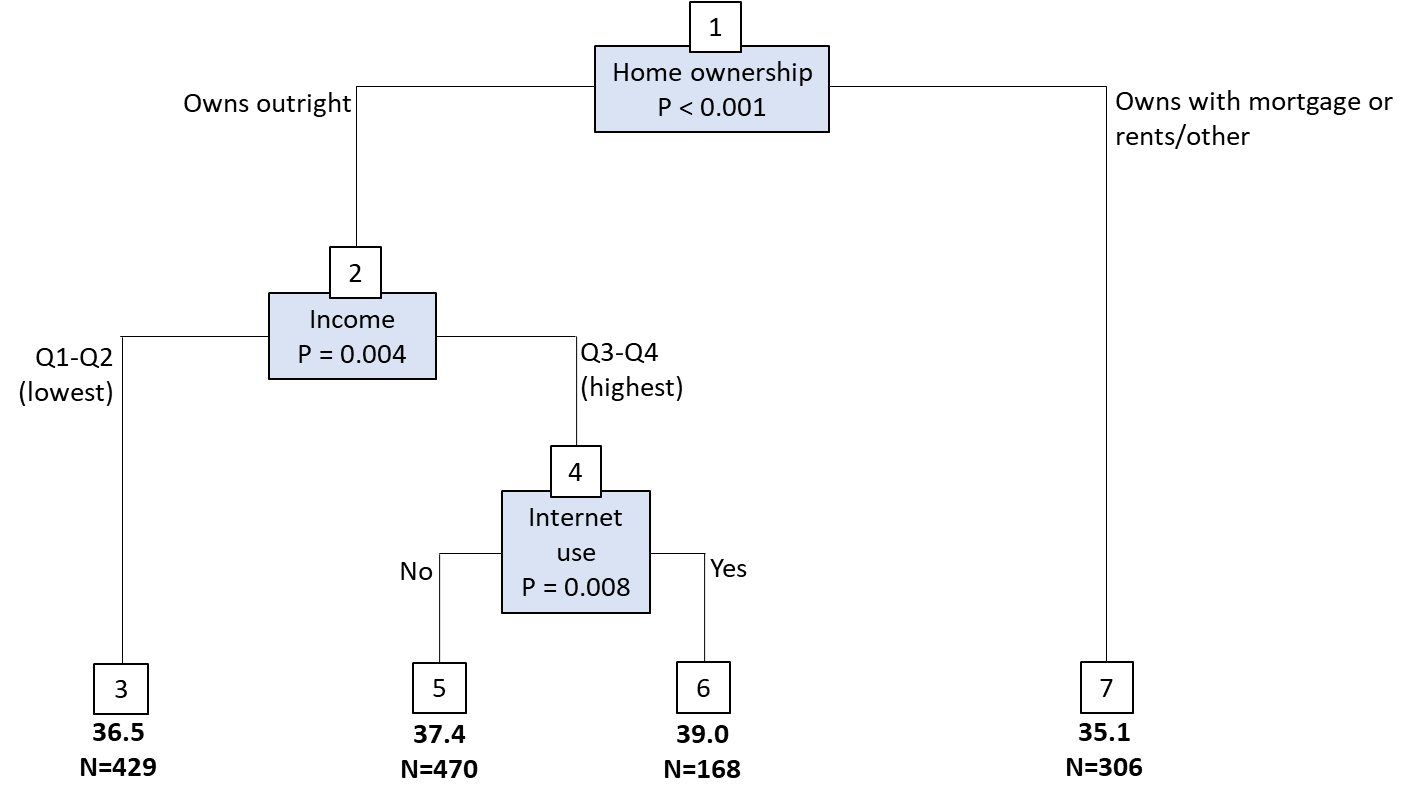


1. CART


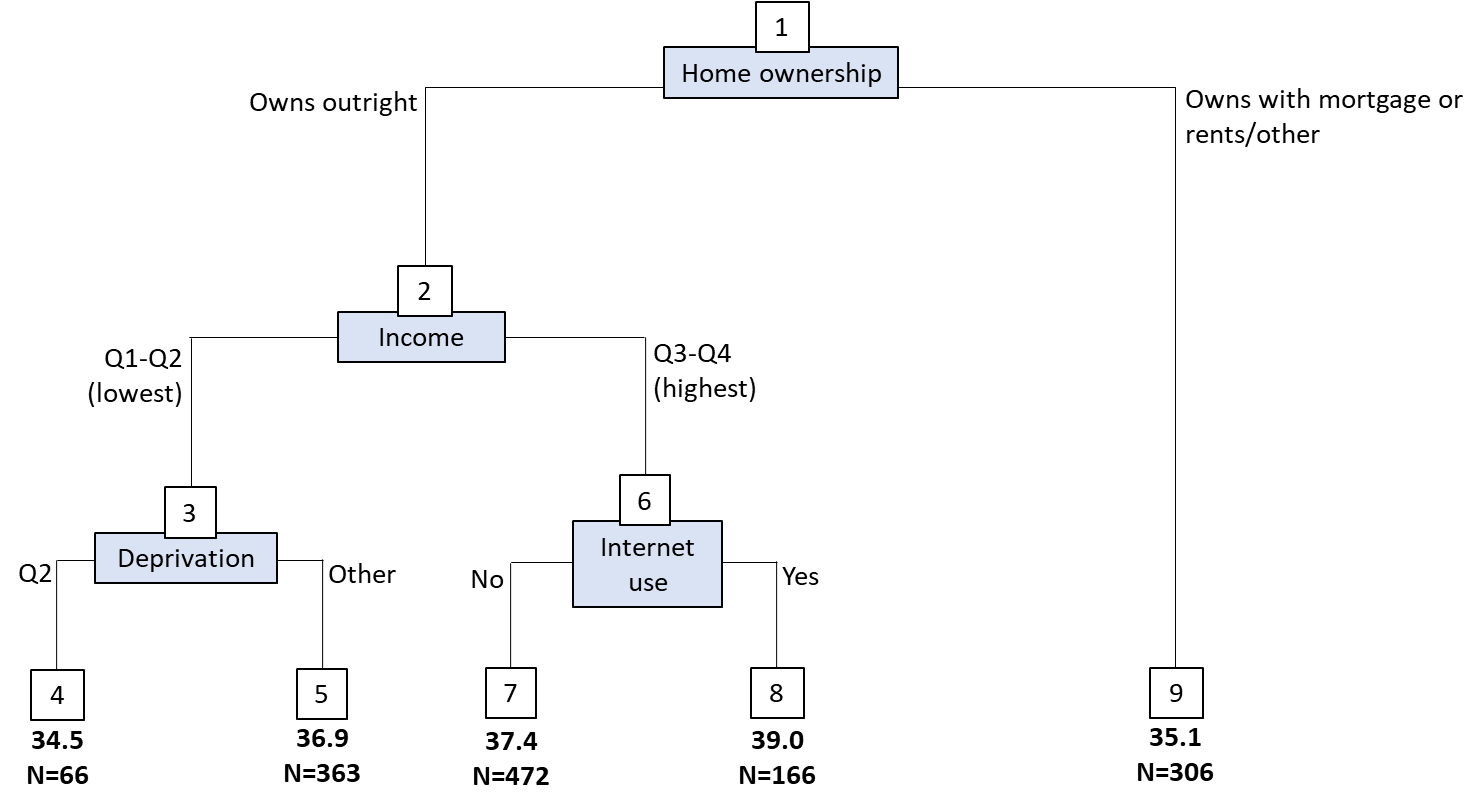


*Note*. The numbers in boxes are the node numbers. The nodes at the bottom of the tree are the terminal nodes, and the mean quality of life scores are reported for each terminal node. Cross-validated R^2^: A) 0.031, B) 0.026.

**Supplementary Figure 4.** Importance of contributing factors for quality of life determined from a random forest model (n=500)


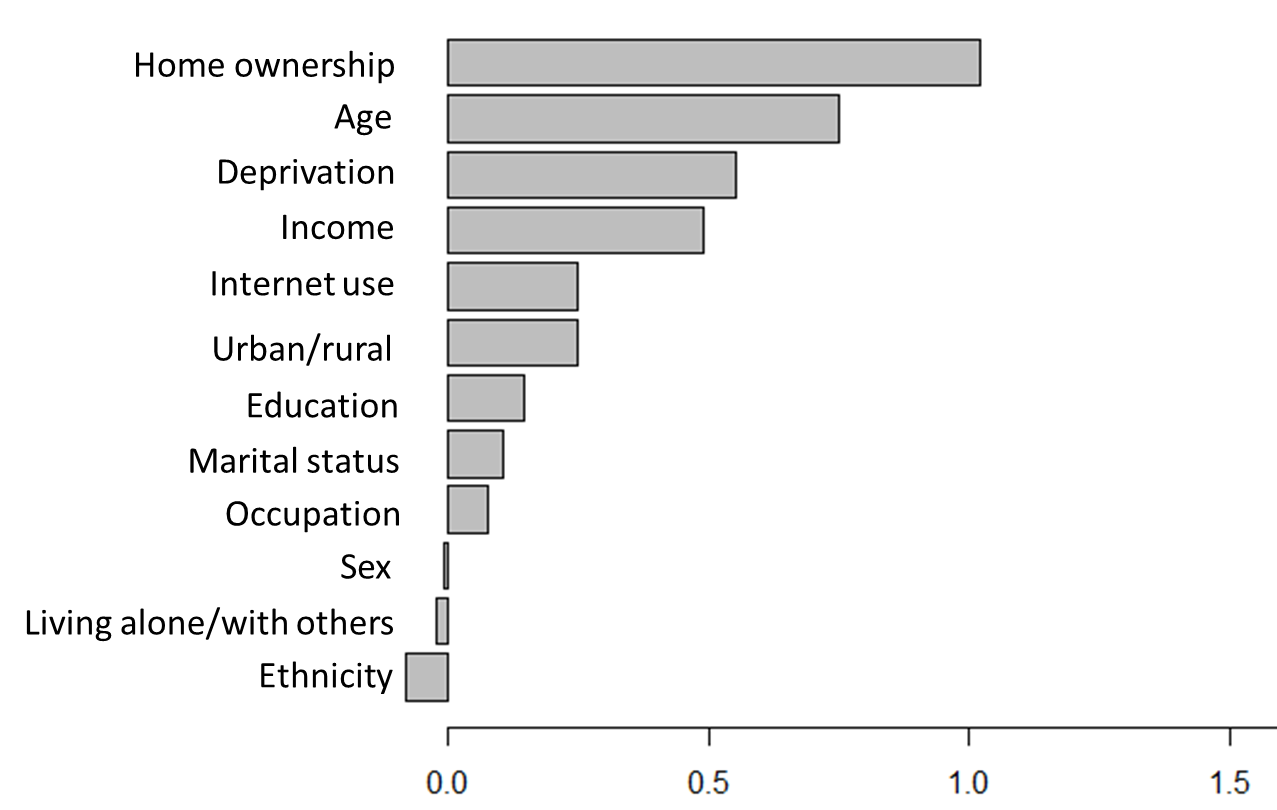


**Supplementary Figure 5**. Regression trees for satisfaction with life and measures of inequality with age removed from the analysis

1. CIT


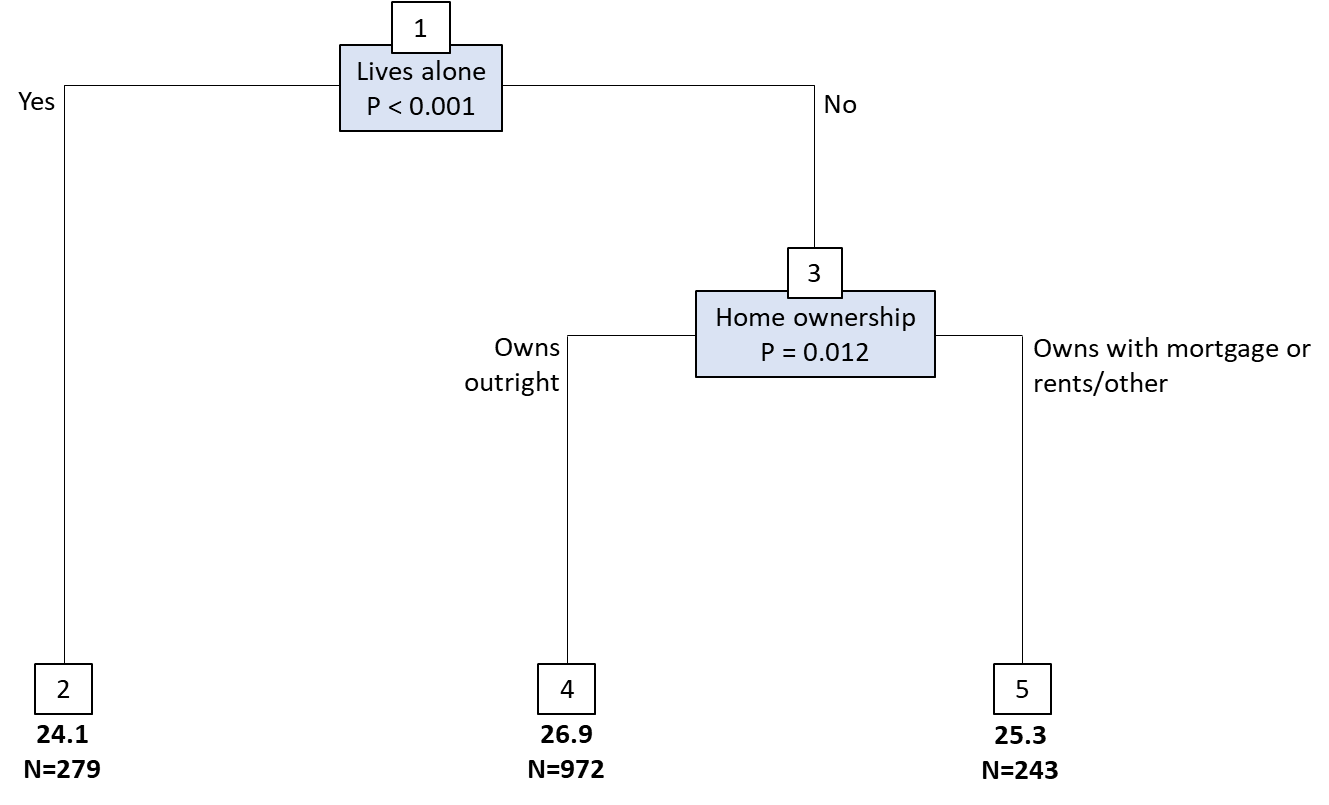


1. CART


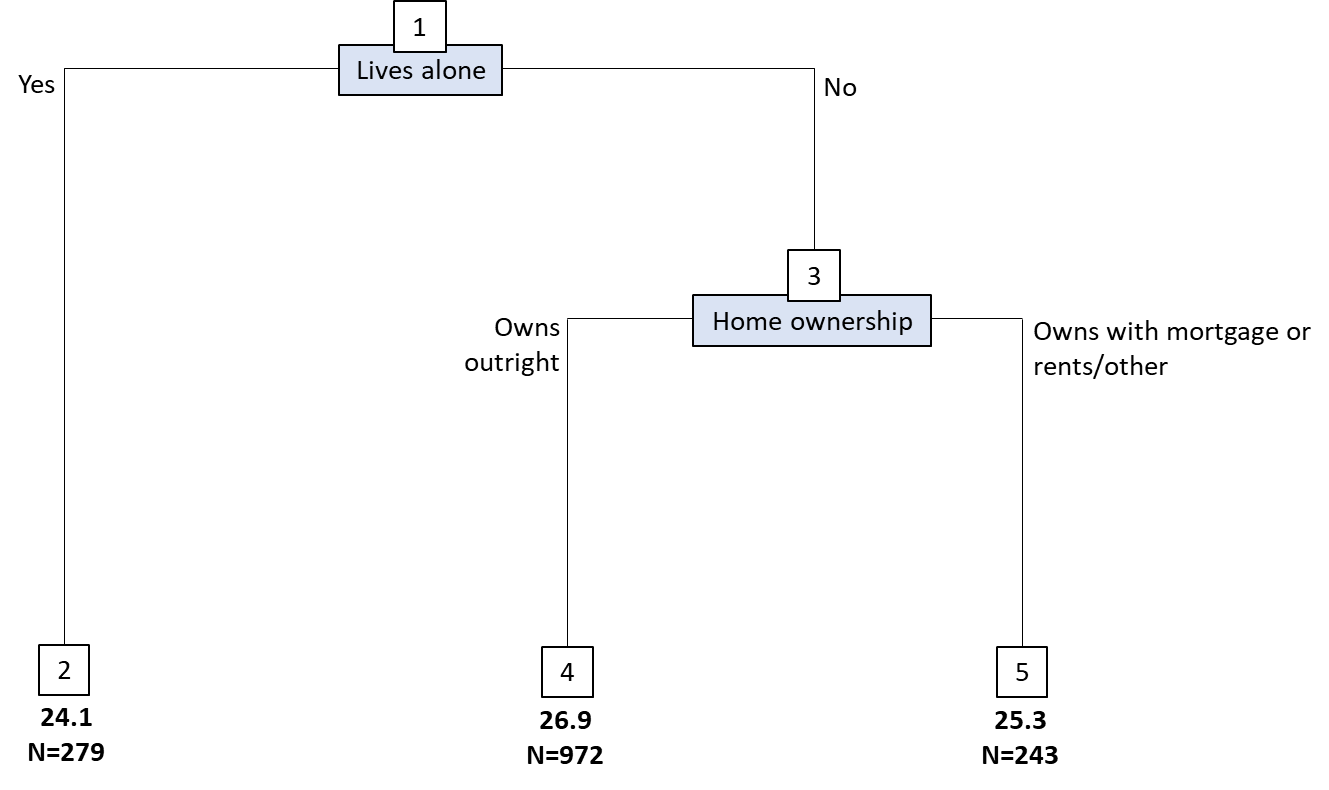


*Note*. The numbers in boxes are the node numbers. The nodes at the bottom of the tree are the terminal nodes, and the mean satisfaction with life scores are reported for each terminal node. Cross-validated R^2^: A) 0.023, B) 0.020.

**Supplementary Figure 6.** Importance of contributing factors for satisfaction with life determined from a random forest model (n=500)


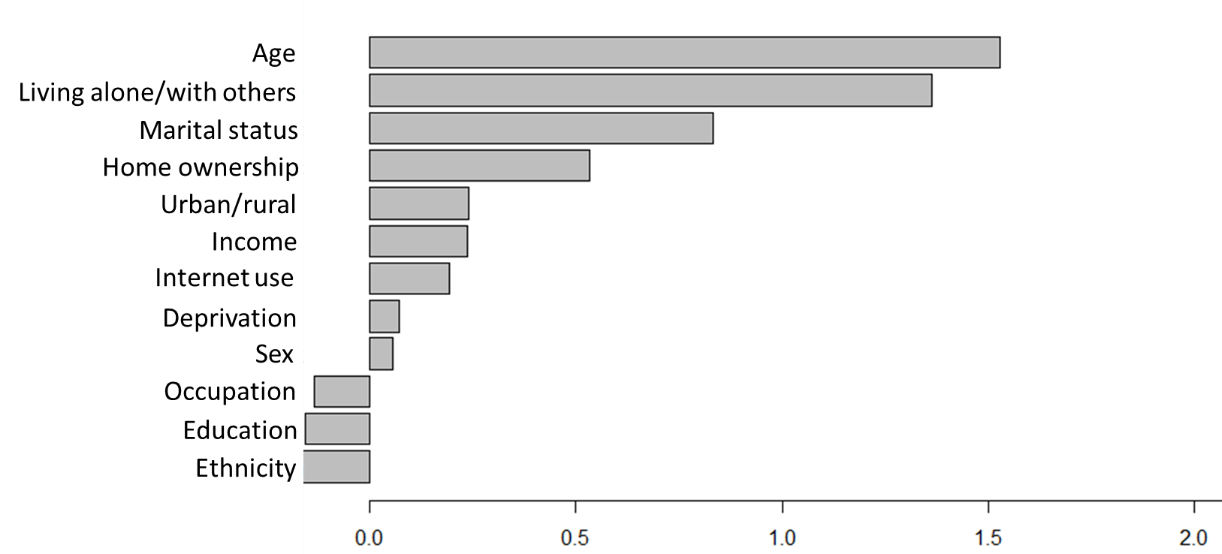


**Supplementary Figure 7.** Regression trees for well-being and measures of inequality with age removed from the analysis

1. CIT


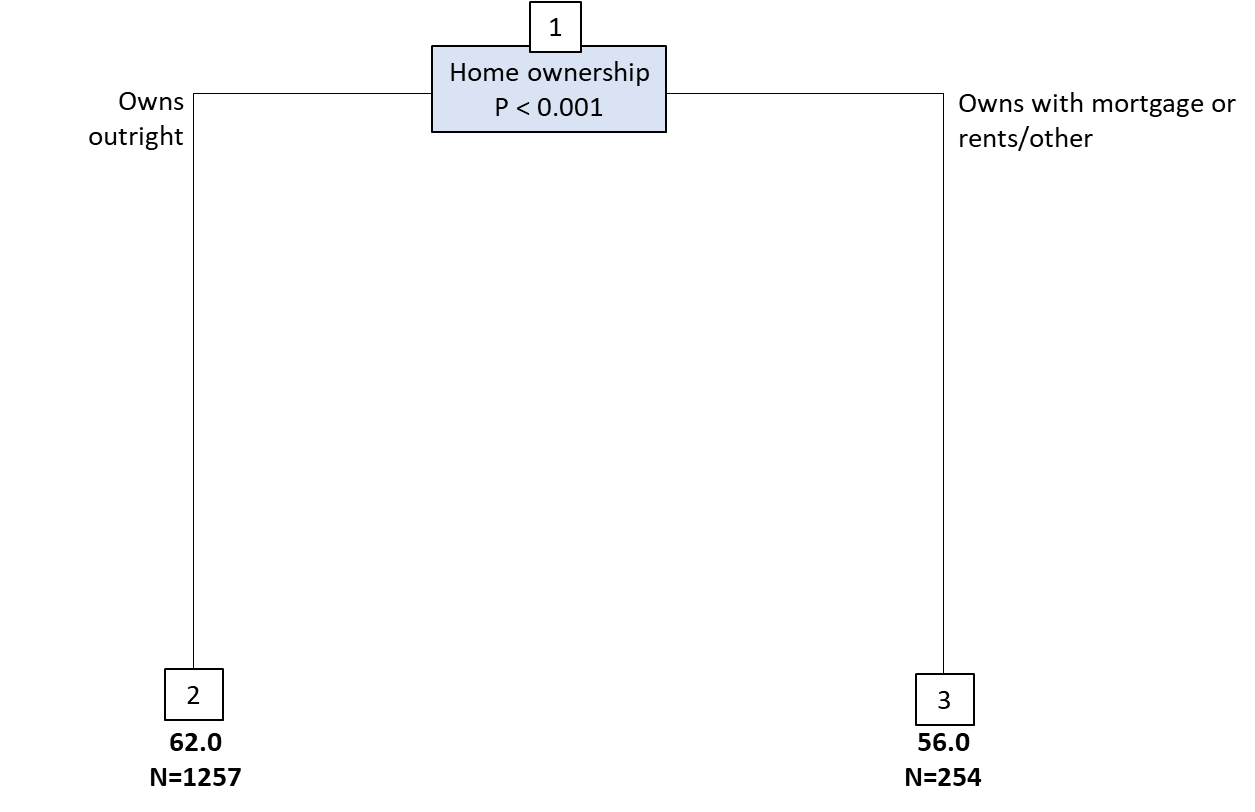


1. CART


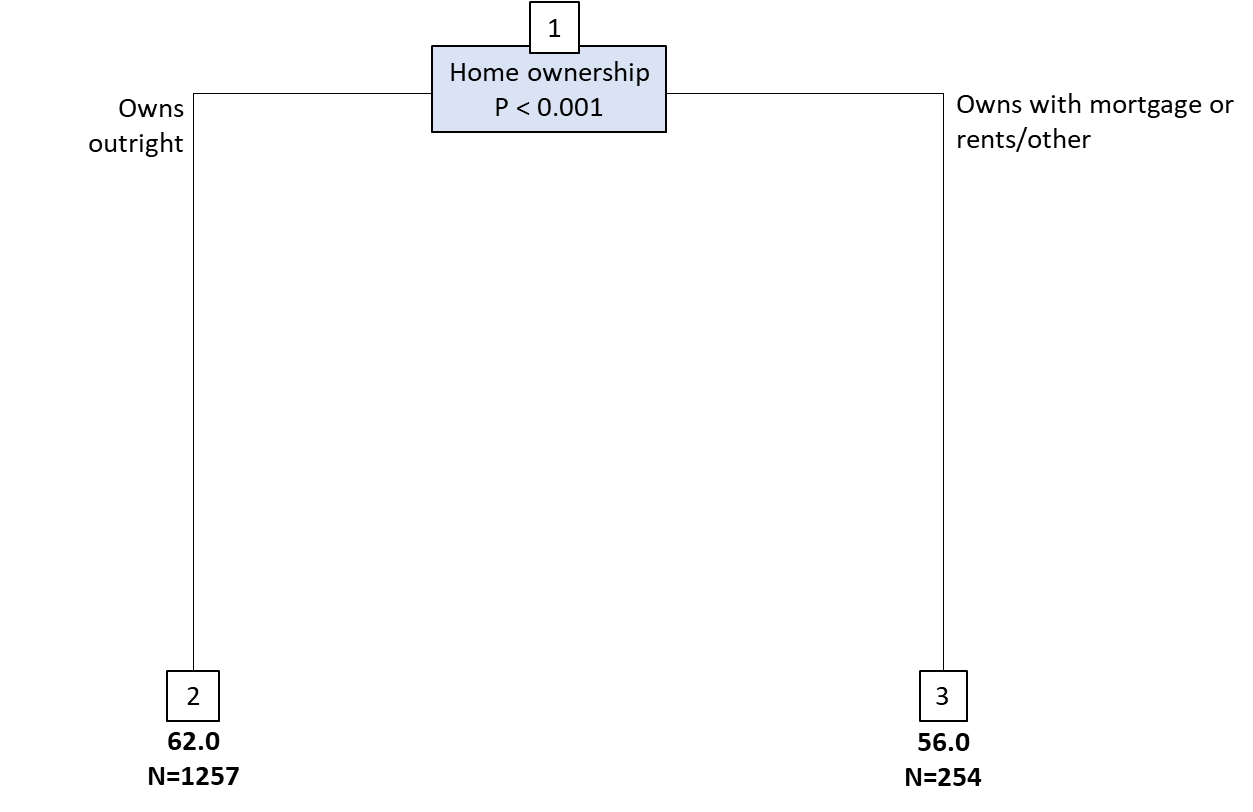


*Note*. The numbers in boxes are the node numbers. The nodes at the bottom of the tree are the terminal nodes, and the mean well-being scores are reported for each terminal node. Cross-validated R^2^: A) 0.005, B) 0.007.

**Supplementary Figure 8.** Importance of contributing factors for well-being determined from a random forest model (n=500)


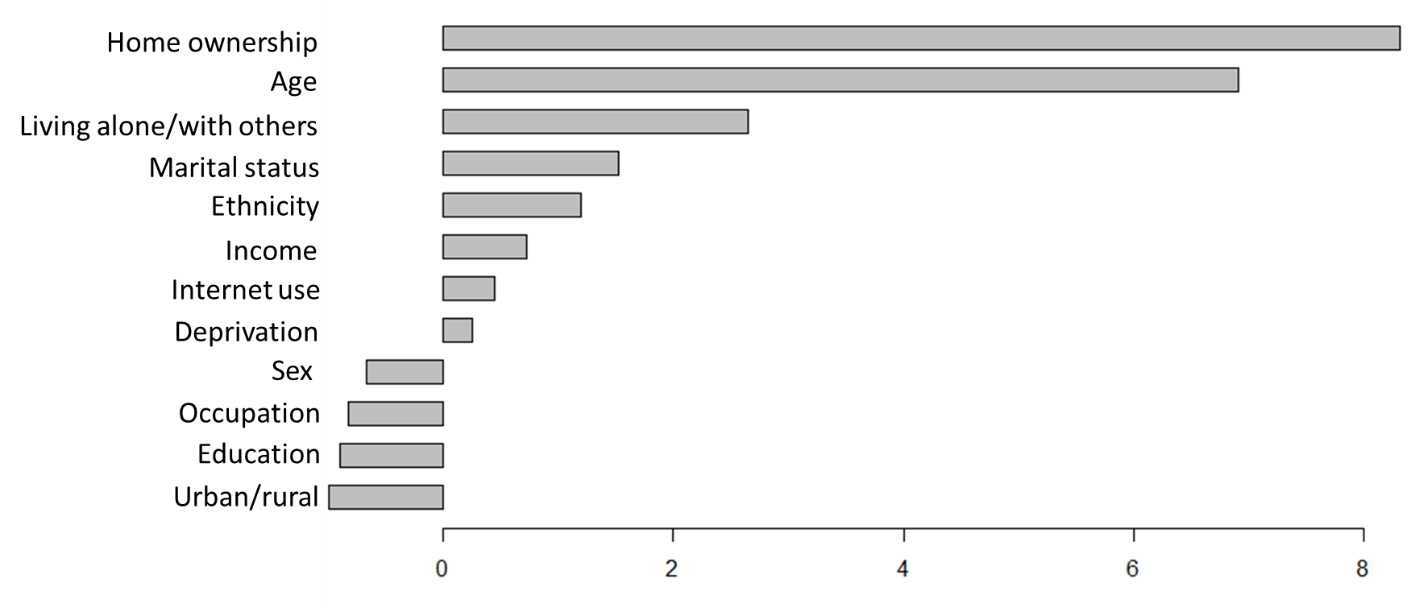


**References**

Breiman, L. (1984). *Classification and Regression Trees*. Chapman & Hall.

Harper, P.R. (2005). A review and comparison of classification algorithms for medical decision making. *Health Policy*, *71*(3), 315–331. <https://doi.org/10.1016/j.healthpol.2004.05.002>

Hothorn, T., Hornik, K., & Zeileis, A. (2006). Unbiased recursive partitioning: a conditional inference framework. *Journal of Computational and Graphical Statistics*, *15*(3), 651–674. <https://doi.org/10.1198/106186006X133933>

Hsieh, S., Schubert, S., Hoon, C., Mioshi, E., & Hodges, J.R. (2013). Validation of the Addenbrooke's Cognitive Examination III in frontotemporal dementia and Alzheimer's disease. *Dementia and Geriatric Cognitive Disorders*, *36*(3-4), 242–250. <https://doi.org/10.1159/000351671>

Lemon, S.C., Roy, J., Clark, M.A., Friedmann, P.D., & Rakowski, W. (2003). Classification and regression tree analysis in public health: methodological review and comparison with logistic regression. *Annals of Behavioral Medicine*, *26*(3), 172–181. <https://doi.org/10.1207/s15324796abm2603_02>

Strobl, C., Malley, J., & Tutz, G. (2009). An introduction to recursive partitioning: rationale, application, and characteristics of classification and regression trees, bagging, and random forests. *Psychological Methods*, *14*(4), 323–348. <https://doi.org/10.1037/a0016973>

Venkatasubramaniam, A., Wolfson, J., Mitchell, N., Barnes, T., JaKa, M., & French, S. (2017). Decision trees in epidemiological research. *Emerging Themes in Epidemiology*, *14*(1), 11. <https://doi.org/10.1186/s12982-017-0064-4>
